# Tumor heterogeneity underlies clinical outcome and MEK inhibitor response in somatic *NF1*-mutant glioblastoma

**DOI:** 10.1172/jci.insight.192658

**Published:** 2025-09-23

**Authors:** Sixuan Pan, Kanish Mirchia, Emily Payne, S. John Liu, Nadeem Al-Adli, Zain Peeran, Poojan Shukla, Jacob S. Young, Rohit Gupta, Jasper Wu, Joanna Pak, Tomoko Ozawa, Brian Na, Alyssa T. Reddy, Steve E. Braunstein, Joanna J. Phillips, Susan Chang, David A. Solomon, Arie Perry, David R. Raleigh, Mitchel S. Berger, Adam R. Abate, Harish N. Vasudevan

**Affiliations:** 1 Department of Radiation Oncology;; 2 Department of Neurological Surgery;; 3 Department of Bioengineering;; 4 Division of Neuropathology, Department of Pathology;; 5 Department of Neuro-Oncology; and; 6 Department of Neurology, University of California, San Francisco (UCSF), San Francisco, California, USA.

**Keywords:** Genetics, Oncology, Brain cancer, Drug screens, Molecular genetics

## Abstract

Tumor suppressor *NF1* is recurrently mutated in glioblastoma, leading to aberrant activation of Ras/rapidly accelerated fibrosarcoma (RAF)/MEK signaling. However, how tumor heterogeneity shapes the molecular landscape and efficacy of targeted therapies remains unclear. Here, we combined bulk and single-cell genomics of human somatic *NF1*-mutant, isocitrate dehydrogenase (IDH) wild-type glioblastomas with functional studies in cell lines and mouse intracranial tumor models to identify mechanisms of tumor heterogeneity underlying clinical outcome and MEK inhibitor response. Targeted DNA sequencing identified CDKN2A/B homozygous deletion as a poor prognostic marker in somatic *NF1*-mutant, but not *NF1* wild-type, glioblastoma. Single-nucleus RNA sequencing of human patient *NF1*-mutant glioblastomas demonstrated that mesenchymal-like (MES-like) tumor cells were enriched for MEK activation signatures. Single-cell RNA-sequencing of mouse intracranial glioblastomas treated with the MEK inhibitor selumetinib identified distinct responses among tumor subpopulations. MEK inhibition selectively depleted MES-like cells, and selumetinib-resistant MES-like cells upregulated Ras signaling while resistant non-MES cells expressed markers of glial differentiation. Finally, genome-wide CRISPR interference screens validated Ras/RAF/MEK signaling as a key mediator of selumetinib response. Repression of the RAF regulator *SHOC2* sensitized glioblastomas to selumetinib in vitro and in vivo, suggesting a synergistic treatment strategy. Taken together, these results highlighted the heterogeneity of *NF1*-mutant glioblastomas and informed future combination therapies.

## Abstract 2

NF1 mutant glioblastomas exhibit prognostic signatures and sensitivity to MEK inhibition

## Graphical abstract

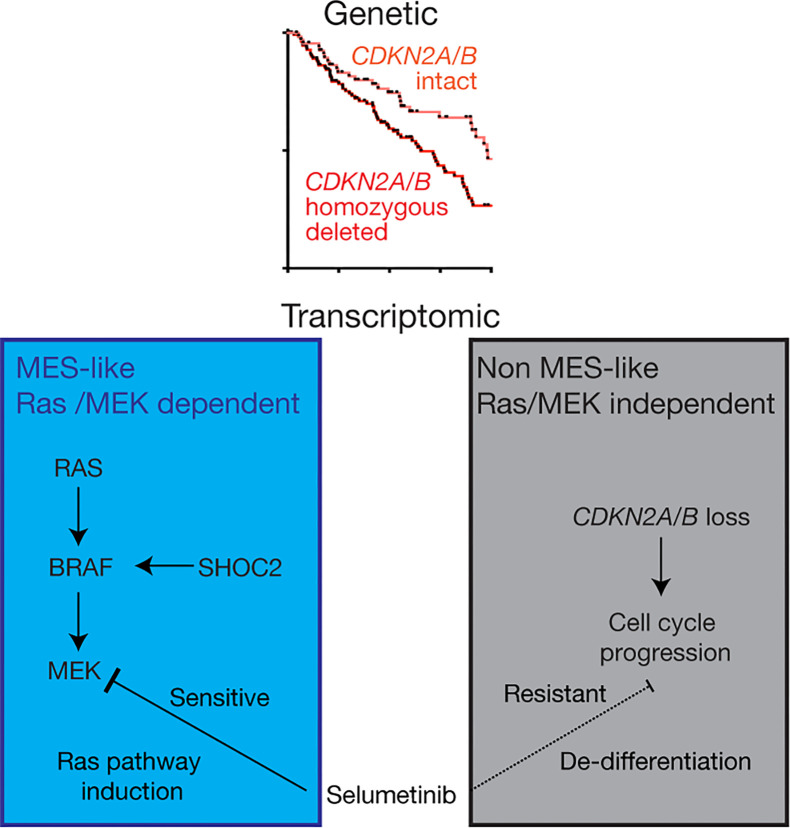

## Introduction

The tumor suppressor *NF1* is mutated in 15% of glioblastomas (1–3), the most common malignant brain tumor with poor outcomes and few effective treatments (4). NF1 is a GTPase-activating protein that negatively regulates Ras, and thus, *NF1* loss leads to induction of Ras/rapidly accelerated fibrosarcoma (RAF)/MEK signaling, driving tumorigenesis and comprising a targetable molecular cascade (5, 6). Genomic studies of glioblastoma have associated *NF1* mutation with enrichment of mesenchymal-like (MES-like) transcriptional tumor cell state and an altered tumor microenvironment (7, 8). More broadly, DNA methylation analysis has identified multiple epigenetic subgroups that overlap with transcriptomic subtypes and DNA alterations, underscoring the complex relationship between genetic drivers and molecular signatures (9). While the updated 2021 WHO Classification of Tumors of the Central Nervous System incorporates an ever-increasing number of molecular criteria for diffuse astrocytic tumors (10), the existence and clinical importance of heterogeneity between and within somatic *NF1*-mutant, isocitrate dehydrogenase (IDH) wild-type glioblastomas remain unclear.

Preclinical studies support MEK inhibition as a selective therapeutic strategy in *NF1*-mutant nervous system tumors including gliomas (11, 12). The MEK inhibitor selumetinib is FDA approved for treating nervous system tumors in patients with syndromic neurofibromatosis type 1 (NF-1) harboring a germline *NF1* mutation (13, 14). In NF-1–associated gliomas, MEK inhibition has demonstrated partial efficacy in *NF1*-mutant gliomas (11, 15), and combined BRAF/MEK inhibition has proven effective in BRAF p.V600E mutant gliomas (16), further supporting the translational potential of Ras/RAF/MEK/ERK blockade within genetically defined glioma subtypes. Nevertheless, treatment resistance to molecular monotherapy remains a challenge (17–20), and mechanisms underlying MEK inhibitor resistance in *NF1*-mutant glioma are unknown.

Here, we combine multimodal molecular and functional analyses to characterize clinically relevant subgroups and mechanisms of MEK inhibition response in *NF1*-mutant, IDH wild-type glioblastomas. Targeted DNA sequencing of *NF1*-mutant, IDH wild-type glioblastomas (*n* = 186) identified *CDKN2A/B* homozygous deletion as a poor prognostic marker in *NF1*-mutant, but not *NF1* wild-type, glioblastomas. Single-nucleus RNA sequencing (snRNA-Seq) of patient glioblastomas (*n* = 9) demonstrated that MES-like cells within *NF1*-mutant tumors exhibited increased MEK activation. Single-cell RNA sequencing (scRNA-Seq) of selumetinib-treated mouse intracranial glioblastomas revealed divergent responses among tumor subpopulations, with MES-like cells displaying Ras pathway induction and non-MES cells exhibiting markers of glial differentiation. Finally, genome-wide CRISPR interference (CRISPRi) screens of selumetinib response highlighted *BRAF* and *SHOC2* as key mediators of MEK inhibitor sensitivity. Repression of *SHOC2* significantly improved selumetinib response both in vitro and in vivo. Taken together, our findings identify clinically important subgroups of *NF1*-mutant, IDH wild-type glioblastomas and delineate mechanisms of MEK inhibitor response, informing the development of synergistic therapies for *NF1*-mutant glioblastomas.

## Results

### Targeted DNA sequencing of somatic NF1-mutant, IDH wild-type glioblastomas identifies CDKN2A/B loss as a negative prognostic factor.

**Figure 1:**
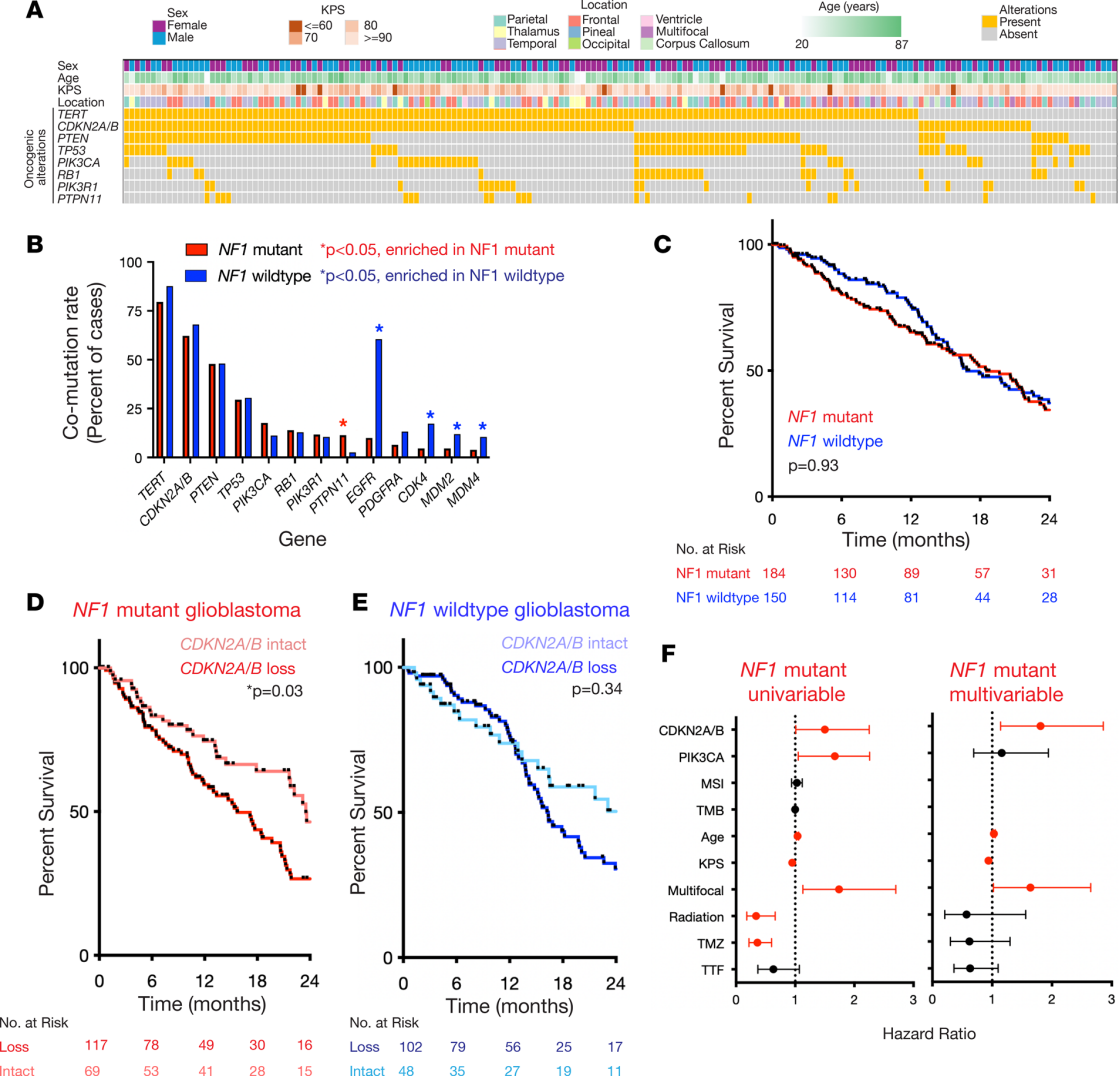
Targeted DNA sequencing of somatic *NF1-*mutant, IDH wild-type glioblastoma (*n* = 186) reveals *CDKN2A/B* codeletion is associated with significantly worse overall survival. (**A**) DNA mutational analysis of *NF1*-mutant, IDH wild-type glioblastoma (*n* = 186) identifies recurrent co-alterations (>10% of tumors) in the *TERT* promoter, *TP53*, cell cycle regulators (*CDKN2A/B*, *RB1*), PI3K signaling (*PTEN*, *PIK3CA*, *PIK3R1*), and Ras signaling (*PTPN11*). (**B**) Analysis of recurrently comutated genes in *NF1*-mutant and propensity score–matched *NF1* wild-type glioblastoma cohort reveals *NF1*-mutant tumors exhibit significantly increased co-alteration with *PTPN11* mutation and are mutually coexclusive with *EGFR*, *CDK4*, *MDM2*, and *MDM4* alterations. (**C**) *NF1*-mutant glioblastomas do not demonstrate significantly different outcomes compared with *NF1* wild-type glioblastomas in a propensity score–matched cohort of IDH wild-type glioblastomas. (**D**) *CDKN2A/B* homozygous loss is associated with significantly worse OS compared with *CDKN2A/B*-intact tumors across *NF1*-mutant (log-rank test, *P* = 0.03) but not (**E**) *NF1* wild-type glioblastomas (log-rank test, *P* = 0.34). (**F**) Multivariable CPH analysis of recurrent genetic alterations shows *CDKN2A/B* loss is the only independent genetic factor significantly associated with worse OS in *NF1*-mutant glioblastoma. Factors in red are significantly associated with OS.

To define the genetic landscape of *NF1*-mutant, IDH wild-type glioblastoma, we retrospectively identified 186 newly diagnosed glioblastomas that underwent targeted DNA sequencing and harbored a somatic *NF1* mutation (Supplemental Table 1; supplemental material available online with this article; https://doi.org/10.1172/jci.insight.192658DS1). Neuropathology review was performed to confirm that all cases met diagnostic criteria for this tumor type using the 2021 WHO Classification of Tumors of the Central Nervous System (10). Cases with a history of NF-1, germline NF1 mutation, or methylation class match to high-grade astrocytoma with piloid features (HGAP) were excluded. Targeted sequencing across *NF1*-mutant, IDH wild-type glioblastomas identified recurrent alterations occurring in at least 10% of tumors, including the *TERT* promoter and genes associated with cell cycle (*CDKN2A/B*, *RB1*, *CDK4*), PI3K signaling (*PTEN*, *PIK3CA*, *PIK3R1*), or apoptosis (*TP53*, *MDM2*, *MDM4*), consistent with prior glioblastoma genomic analysis (Figure 1A) (1–3). Compared with a propensity score–matched *NF1* wild-type, IDH wild-type glioblastoma cohort (Supplemental Table 2), *NF1*-mutant glioblastomas were significantly more likely to harbor comutation of *PTPN11*, an upstream Ras regulator, while *NF1* wild-type glioblastomas were significantly more likely to harbor *EGFR* amplification and amplification of cell cycle/apoptosis–associated oncogenes *CDK4*, *MDM2*, or *MDM4* (Figure 1B). No significant difference in overall survival (OS) was observed between *NF1*-mutant and *NF1* wild-type glioblastomas (Figure 1C), and no significant differences were observed with respect to sex, age, or Karnofsky Performance Status (KPS) (Supplemental Figure 1). Cox proportional hazards (CPH) analysis of recurrently comutated genes revealed *CDKN2A/B* homozygous loss was associated with worse OS in *NF1*-mutant but not *NF1* wild-type glioblastomas (Figure 1, D and E, and Supplemental Table 3). CPH analysis of clinical variables associated with OS in the *NF1*-mutant, IDH wild-type glioblastoma cohort revealed age, KPS (21, 22), multifocality, adjuvant radiation (23, 24), adjuvant temozolomide (25, 26), and tumor-treating fields (27) as being associated with OS (*P* < 0.1) (Figure 1F). Consistent with this observation, analysis of The Cancer Genome Atlas IDH wild-type glioblastoma cohort similarly showed *NF1*-mutant, *CDKN2A/B*-deleted tumors were associated with worse OS than *NF1*-mutant, *CDKN2A/B*-intact tumors (Supplemental Figure 2A). With regard to baseline clinical factors, *CDKN2A/B* deletion was not associated with differences in patient sex, age, or KPS compared with *CDKN2A/B*-intact glioblastomas (Supplemental Figure 2, B–D). On multivariable CPH analysis incorporating significant clinical variables, *CDKN2A/B* loss was the only genetic co-alteration that remained significantly associated with worse OS in *NF1*-mutant glioblastoma (Figure 1F and Supplemental Table 4). Taken together, DNA mutational analysis suggests *NF1*-mutant and *NF1* wild-type tumors exhibit differences in comutation patterns with no global difference in baseline clinical parameters or outcomes, and *CDKN2A/B* loss is a negative prognostic marker in *NF1*-mutant but not *NF1* wild-type glioblastomas, which underscores the intertumor heterogeneity across *NF1*-mutant, IDH wild-type glioblastomas.

### SnRNA-Seq of human NF1-mutant glioblastoma reveals MES-like cell state correlates with Ras/RAF/MEK activation.

**Figure 2:**
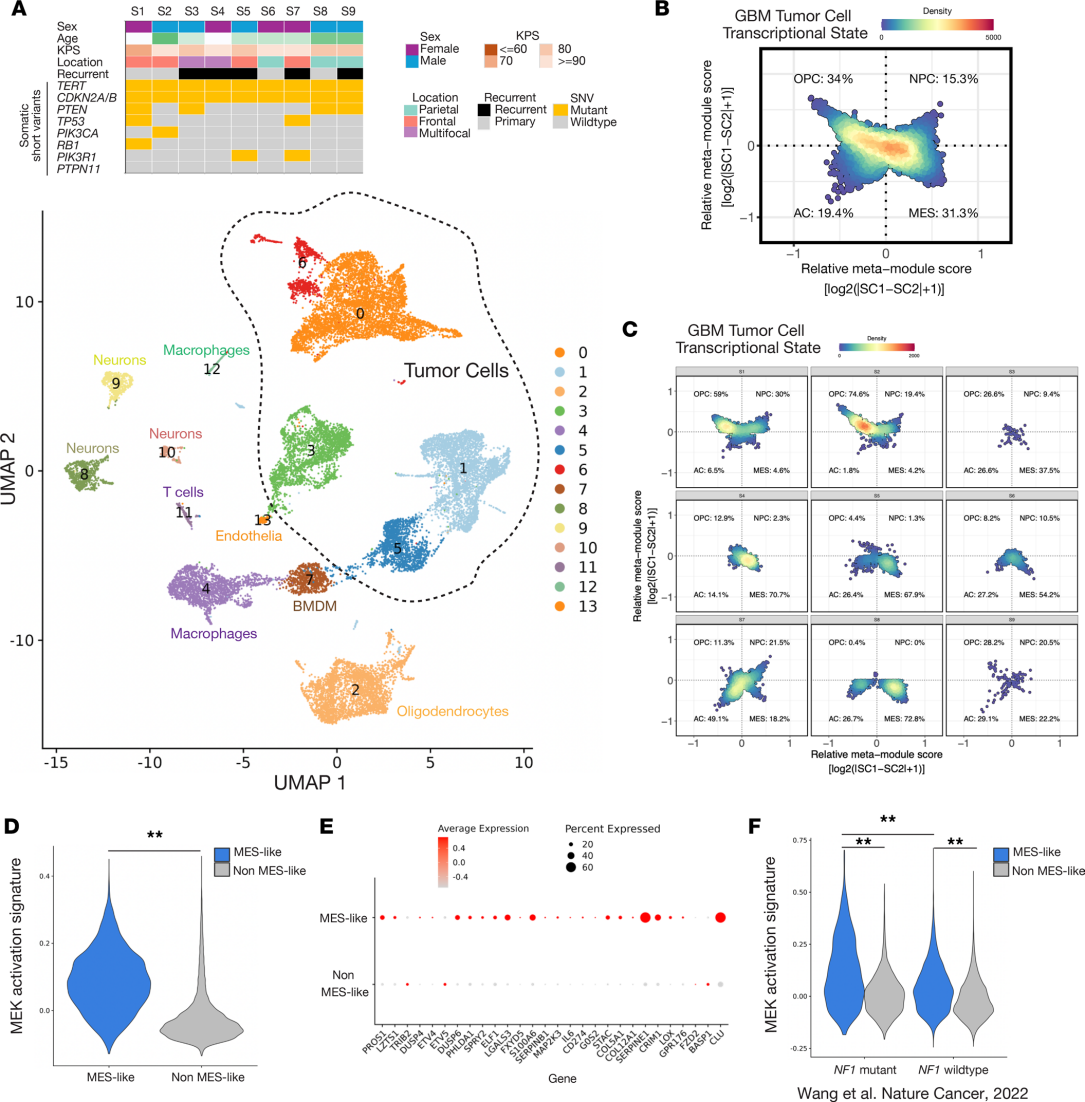
SnRNA-Seq of human *NF1*-mutant, IDH wild-type glioblastoma (*n* = 9) reveals MES-like cells exhibit Ras pathway activation. (**A**) SnRNA-Seq of 21,959 nuclei from 9 patient-derived human *NF1*-mutant, IDH wild-type glioblastomas identifies 5 tumor cell clusters and 9 nontumor cell clusters. (**B**) Tumor cells were distributed across 4 cell states (OPC-like, NPC-like, AC-like, MES-like), with relative enrichment for MES-like and OPC-like cell states. (**C**) Individual *NF1*-mutant glioblastomas samples are divided into MES-like enriched (samples S3, S4, S5, S6, and S8) and non-MES enriched (sample S1, S2, S7, S9) tumors. (**D**) MES-like cells express significantly increased MEK activation compared with non-MES cells (** *P* < 0.001, Wilcoxon rank-sum test). (**E**) Single-cell dot plot expression analysis of 31 genes comprising the MEK activation gene set confirms enrichment in MES-like compared with non-MES cells. (**F**) Integration with published glioblastoma snRNA-Seq data (45) comparing *NF1*-mutant and *NF1* wild-type tumors reveals the MEK activation signature is significantly enriched in MES-like cells within *NF1*-mutant glioblastomas compared with both non-MES cells in *NF1*-mutant glioblastomas and MES-like cells in *NF1* wild-type glioblastomas (** *P* < 0.001, Wilcoxon rank-sum test). UMAP, uniform manifold approximation and projection; SNV, single nucleotide variant.

In addition to intertumor heterogeneity, glioblastomas exhibit substantial intratumor heterogeneity defined by 4 cell states: MES-like, neural progenitor cell–like (NPC-like), oligodendrocyte progenitor-like (OPC-like), and astrocyte-like (AC-like) (7). Prior work has demonstrated *NF1*-mutant glioblastomas are enriched for MES-like transcriptional signatures compared with *NF1* wild-type glioblastomas, but how the cellular composition varies across *NF1*-mutant glioblastomas and the dominant signaling mechanisms and potential druggable dependencies within the MES-like versus non-MES compartments remain unclear. To investigate the intratumor heterogeneity within somatic *NF1*-mutant, IDH wild-type glioblastomas, we next performed snRNA-Seq on a total of 21,959 nuclei from 9 patient glioblastomas (Figure 2A). Integrated UMAP revealed a total of 14 cell clusters comprising 5 tumor cell clusters and 9 nontumor cell clusters defined by *PTPRC* (CD45), marker gene expression, and copy number alterations (CNAs) (Figure 2A; Supplemental Figure 3, A–E; and Supplemental Table 5) (28, 29). Tumor cells showed recurrent whole chromosome 7 gain, whole chromosome 10 loss, chromosome 13q loss, or chromosome 19 gain (Supplemental Figure 3E). Nontumor cell clusters included oligodendrocytes (C2), macrophages (C4, C12), bone marrow–derived macrophages (C7), neurons (C8, C9, C10), T lymphocytes (C11), and endothelial cells (C13) (Supplemental Figure 4, A–C, and Supplemental Table 6). We first analyzed glioblastoma tumor cells, which we classified into MES-like, NPC-like, OPC-like, or AC-like cell states based on published cell-scoring methods (7). In aggregate, *NF1*-mutant glioblastomas were composed of an admixture combining all 4 transcriptional cell states, and OPC-like and MES-like cells were more common than NPC-like or AC-like cells (Figure 2B). Multiple subclones were identified within each tumor sample (Supplemental Figure 5A), with OPC-like and NPC-like cells more likely to be cycling, while MES-like cells were least likely to be cycling as previously reported (Supplemental Figure 5B) (7). On a per-sample basis, MES-like cells were the dominant tumor cell state in 5/9 (55%) tumors while the remaining 4 tumors were enriched for different non-MES subpopulations (Figure 2C). Given *NF1* loss leads to Ras/RAF/MEK misactivation, we next sought to better understand which cellular states exhibited transcriptional signatures of Ras/REF/MEK activation. MES-like cells in *NF1*-mutant glioblastomas demonstrated significantly increased expression of transcriptional MEK activation and MEK/ERK target gene signatures compared with non-MES cells (Figure 2, D and E, and Supplemental Figure 5, C and D) (30, 31). Taken together, these data suggest that while MES-like cells are enriched within *NF1*-mutant glioblastomas, marked inter- and intratumor heterogeneity exists, and MES-like cells harbor distinct transcriptional signatures enriched for Ras/RAF/MEK activation downstream of *NF1* mutation (28, 29).

To more broadly understand how *NF1* mutation affects glioblastoma cellular populations compared with *NF1* wild-type glioblastomas, we next analyzed published glioblastoma snRNA-Seq data comprising both *NF1*-mutant and *NF1* wild-type tumors (28, 29). UMAP analysis revealed a total of 22 cell clusters comprising 10 tumor cell clusters and 12 nontumor cell clusters that were generally conserved based on *NF1* mutation status or sample of origin (Supplemental Figure 6, A–D, and Supplemental Table 7). The 12 nontumor cell clusters broadly comprised oligodendrocytes (clusters 1, 2, 13, 19), microglia/macrophages (clusters 3, 10, 12, 17, 18), neurons (clusters 8 and 21), and T/NK cells (cluster 15) (Supplemental Figure 6, E and F), consistent with reported glioblastoma microenvironmental subpopulations (28, 29, 32). Given prior studies suggest *NF1*-mutant glioblastomas harbor a distinct microenvironment (8), we first compared tumor microenvironment cell clusters between *NF1*-mutant and *NF1* wild-type samples. *NF1*-mutant glioblastomas had significantly fewer neurons (C8), but no other nontumor populations existed in significantly different proportions between *NF1*-mutant and *NF1* wild-type glioblastomas (Supplemental Figure 7A). Gene set enrichment analysis (GSEA) of differentially expressed genes within other nontumor cell clusters revealed *NF1*-mutant tumors upregulated pro-inflammatory interferon signaling pathways in oligodendrocytes (C1, C2) but downregulated these same pathways in microglia/macrophages (C12), underscoring the complex associations between *NF1* mutation and microenvironmental composition (Supplemental Figure 7B). We next analyzed differences in tumor cell state assigned to 4 main cell types (OPC-like, NPC-like, AC-like, MES-like) using the published cell state–scoring method (Supplemental Figure 8A) (7). Comparing glioblastoma tumor cell states in *NF1*-mutant to *NF1* wild-type glioblastomas, no significant differences in the proportion of tumor cells in a given cell state was observed, though a trend toward increased MES-like cells in *NF1*-mutant tumors was noted as previously described (Supplemental Figure 8B) (2, 7). Although MES-like cells from both *NF1*-mutant and *NF1* wild-type glioblastomas showed increased expression of the MEK activation signature, MES-like cells in *NF1*-mutant glioblastomas showed significantly increased MEK activation compared with MES-like cells in *NF1* wild-type tumors (Figure 2F and Supplemental Figure 8C). Taken together, our analysis reveals *NF1*-mutant MES-like glioblastoma tumor cells harbor a distinct molecular signature associated with Ras/RAF/MEK activation compared with either non-MES cells or *NF1* wild-type glioblastomas.

### Different tumor cell subpopulations exhibit distinct transcriptional signatures underlying selumetinib response in SB28 glioblastoma intracranial tumor models.

Given the increased MEK activation observed in MES-like tumor cells from *NF1*-mutant glioblastomas and the clinical approval of multiple MEK inhibitors for *NF1*-mutant nervous system tumors (13, 33, 34), we next sought to functionally evaluate the therapeutic relevance of MEK inhibition. We tested the efficacy of the MEK inhibitor selumetinib across 9 glioblastoma cell lines, including 6 *NF1*-mutant and 3 *NF1* wild-type cell lines. Consistent with prior work demonstrating MEK inhibitor efficacy selectively against *NF1*-mutant glioblastoma (35, 36), in vitro cell viability analysis showed that *NF1*-mutant glioblastoma cells were significantly more sensitive to selumetinib compared with *NF1* wild-type glioblastoma cells (Supplemental Figure 9). Consistent with this observation, selumetinib significantly extended survival in both *NF1*-mutant GBM43 and *Nras*-mutant SB28, but not *NF1* wild-type GBM6, intracranial tumors in vivo (Supplemental Figure 10, A–C). However, tumors ultimately developed resistance, and the therapeutic effect was not durable.

**Figure 3:**
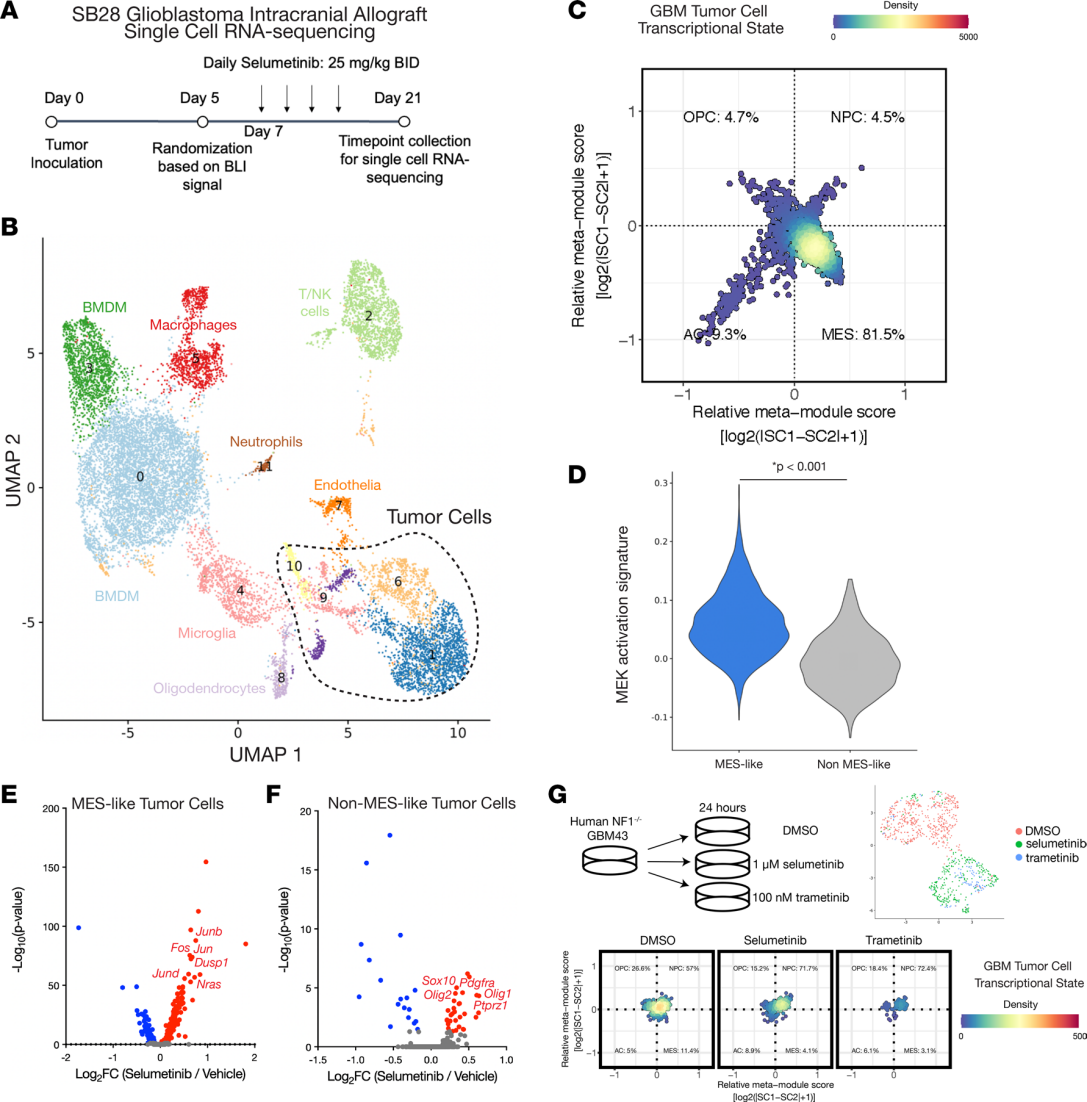
ScRNA-Seq of SB28 intracranial allografts and GBM43 glioblastoma neurospheres reveals distinct transcriptional signatures and selumetinib responses in MES-like compared with non-MES tumor cell subpopulations. (**A**) Experimental overview of scRNA-Seq of selumetinib- versus vehicle-treated SB28 intracranial allografts. BLI, bioluminescence imaging. (**B**) Classification of 3,411 cells as tumor cells. (**C**) Tumor cells were distributed across 4 cell states (OPC-like, NPC-like, AC-like, MES-like). (**D**) Selumetinib-treated MES-like tumor cells show significantly increased expression of an MEK activation signature (* *P* < 0.001, Wilcoxon rank-sum test). (**E**) Selumetinib-treated MES-like cells show significantly increased expression of Ras pathway transcriptional targets, such as *Fos*, *Jun*, *Junb*, *Jund*, and *Dusp1*. (**F**) Selumetinib-treated non-MES cells show significantly increased expression of glial differentiation genes, such as *Olig1*, *Olig2*, *Sox10*, *Ptprz1*, and *Pdgfra*. (**G**) ScRNA-Seq of human GBM43 neurospheres treated with vehicle DMSO, selumetinib, or trametinib reveals depletion of MES-like cells following treatment with either the MEK inhibitor selumetinib or trametinib compared with DMSO control.

The transient therapeutic responses to MEK inhibition raised the possibility of heterogeneous cellular response. To test the hypothesis that tumor heterogeneity underlies the limited efficacy of selumetinib, we next performed scRNA-Seq on a total of 15,604 cells from SB28 intracranial tumors treated with either vehicle (*n* = 3) or selumetinib (*n* = 3) (Figure 3A and Supplemental Figure 11, A–C). Integrated UMAP revealed 12 cell clusters comprising 4 tumor cell clusters and 8 nontumor cell clusters (Figure 3B; Supplemental Figure 11, D–H; and Supplemental Table 8). Differential gene expression and GSEA comparing selumetinib-treated and vehicle-treated conditions across nontumor cell clusters revealed selumetinib treatment was associated with an interferon-mediated inflammatory response in microglia (C4), endothelial cells (C7), and oligodendrocytes (C8), suggesting MEK inhibition may have a pro-inflammatory effect on the glioblastoma tumor microenvironment (Supplemental Figure 11I). Tumor cell transcriptional states’ assignment to 4 cell states (OPC-like, NPC-like, AC-like, MES-like) using a published cell state–scoring method (7) revealed that tumor cell type composition was not significantly different between treatment conditions, and cycling cells were overrepresented in the OPC-like state as previously reported (Figure 3C and Supplemental Figure 12, A–C) (7). The transcriptional MEK activation signature was significantly increased in MES-like compared with non-MES cells (Figure 3D and Supplemental Figure 12D), consistent with human *NF1*-mutant glioblastoma snRNA-Seq analysis. Finally, to understand mechanisms associated with selumetinib resistance, we performed differential expression analysis between selumetinib-treated and vehicle-treated tumor cells (Supplemental Table 9). Selumetinib-treated MES-like cells exhibited Ras pathway activation evidenced by significantly increased expression of the Ras transcriptional targets *Fos*, *Jun*, *Junb*, *Jund*, and *Dusp1* (Figure 3E and Supplemental Figure 12E). In contrast, non-MES cells treated with selumetinib were associated with induction of glial differentiation genes, such as *Olig1*, *Olig2*, *Sox10*, *Ptprz1*, and *Pdgfra* (Figure 3F and Supplemental Figure 12F), though it is possible this difference could be explained by selection of a clonal subpopulation rather than activation of a differentiation program in non-MES glioblastoma cells. In sum, these data suggest distinct mechanisms mediate selumetinib response across different glioblastoma cellular states, with MES-like cells maintaining dependence on Ras/RAF/MEK/ERK signaling while non-MES cells are associated with altered glial differentiation to persist following selumetinib treatment.

To complement this analysis and define the response to MEK inhibition in an orthogonal *NF1*-mutant glioblastoma model, we performed scRNA-Seq of human GBM43 cells treated with vehicle DMSO, selumetinib, or a second MEK inhibitor, trametinib (Figure 3G). GBM43 cells treated with selumetinib or trametinib clustered together, and the proportion of MES-like cells decreased following treatment with either MEK inhibitor compared with vehicle DMSO control (Supplemental Figure 13A and Supplemental Table 10). MES-like cells that persisted following MEK inhibition exhibited Ras pathway activation as evidenced by significantly increased *JUND* and *NRAS* expression (Supplemental Figure 13B). In sum, our single-cell analyses support a model in which MEK activation is enriched in *NF1*-mutant MES-like cells with resistance arising due to compensatory Ras/RAF/MEK activation.

### Genome-wide CRISPRi screens in glioblastoma cells demonstrate conserved mechanisms underlying cell growth and response to the MEK inhibitor selumetinib.

**Figure 4:**
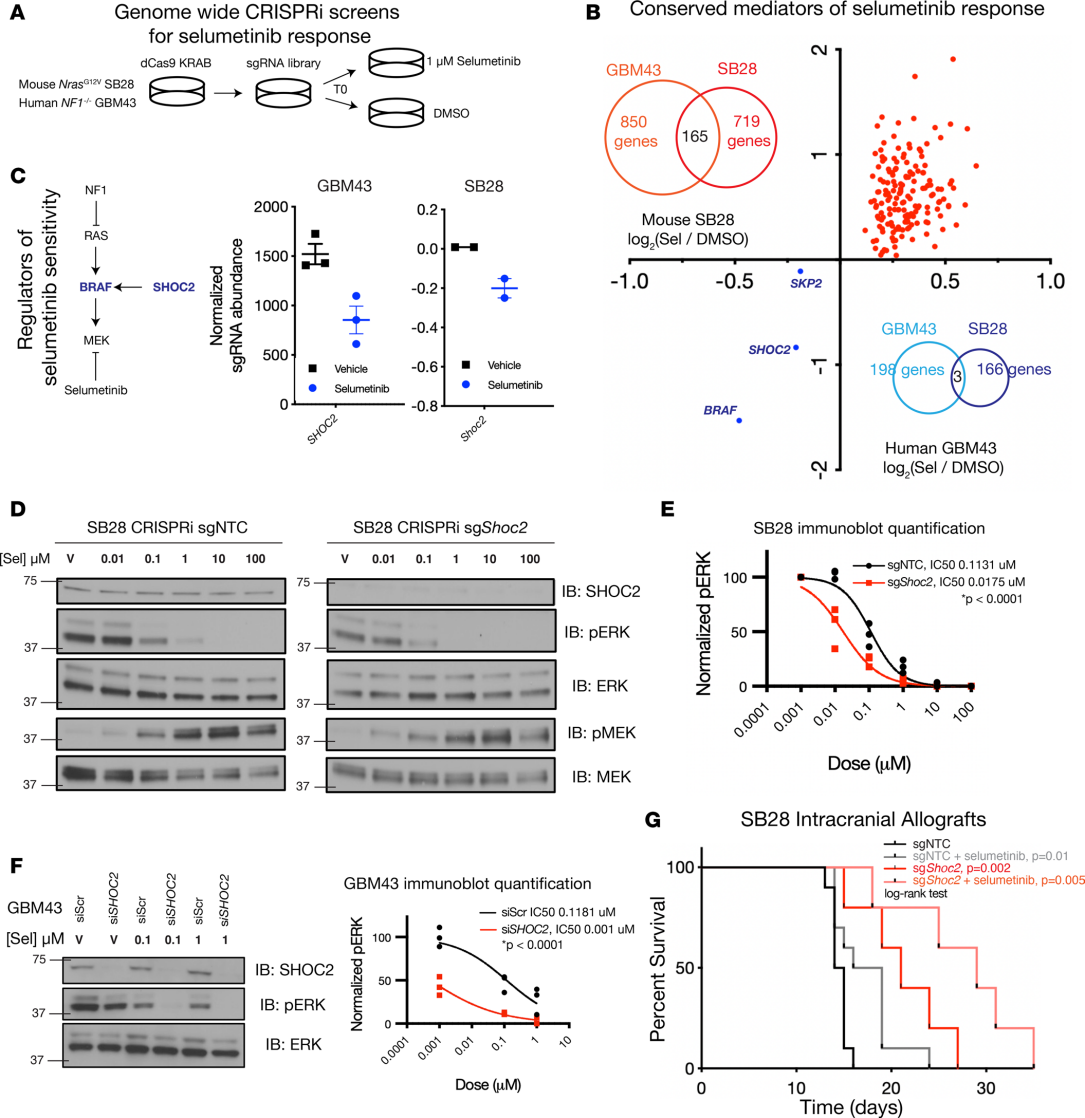
Genome-wide CRISPRi screens in glioblastoma cells reveal cell cycle genes are required for growth and nominate *SHOC2* as a critical mediator of MEK inhibitor selumetinib. (**A**) Schematic of genome-wide CRISPRi screens in *NF1*-mutant human GBM43 (*n* = 3) and *NRAS^G12V^* mouse SB28 (*n* = 2) glioblastoma cells treated with 1 μM selumetinib or vehicle DMSO. (**B**) Integrated analysis of conserved genes significantly modulating glioblastoma cell response to the MEK inhibitor selumetinib (Sel/DMSO) in both SB28 and GBM43 screens (*n* = 165 genes mediating resistance, *n* = 3 genes mediating sensitivity) reveals (**C**) repression of the Ras downstream targets *BRAF* or *SHOC2* mediates selumetinib sensitivity. (**D** and **E**) sg*Shoc2-*deficient SB28 glioblastoma cells (IC_50_ 0.0175, 95% CI 0.011–0.027) are more sensitive to selumetinib than sgNTC SB28 glioblastoma cells (IC_50_ 0.1131, 95% CI 0.080–0.169) (*P* < 0.0001, *F* test). (**F**) siRNA *SHOC2*-deficient GBM43 cells (IC_50_ 0.001; 95% CI 0.0001–0.001) are more sensitive to selumetinib than siScr GBM43 cells (IC_50_ 0.1181; 95% CI 0.04–0.33) (*P* < 0.0001, *F* test). (**G**) CRISPRi *Shoc2* repression is sufficient to improve selumetinib response in SB28 intracranial allografts in vivo (*P* < 0.05, log-rank test, all curves compared with sgNTC condition).

We next sought to identify genetic modulators and potential therapeutic targets underlying selumetinib response using functional screens. We performed genome-wide CRISPRi screens in 2 cell models of *NF1*-mutant glioblastoma: *NF1*-mutant human GBM43 cells and *Nras^G12V^* mutant mouse SB28 cells using a selumetinib dose of 1 μM, which approximates the selumetinib IC_50_ in both GBM43 and SB28 cells, and terminating each screen at an average of 5 population doublings across selumetinib replicates for each cell line (Figure 4A and Supplemental Tables 11 and 12). Given the primary effect of *NF1* loss is Ras misactivation, we rationalized that conserved genetic perturbations mediating growth or MEK inhibitor response across both *NF1*-mutant human GBM43 and *Nras*-mutant mouse SB28 models would reflect robust and reproducible mechanisms underlying *NF1*-mutant glioblastoma biology. Three distinct experimental conditions and time points were assayed in each CRISPRi screen: T0 corresponding to the initial sgRNA distribution prior to any drug treatment, DMSO corresponding to treatment with vehicle that constitutes a control condition to identify genetic perturbations mediating cell growth, and selumetinib corresponding to treatment with 1 μM selumetinib that constitutes the drug treatment condition to identify genetic perturbations mediating selumetinib response. Genes with sgRNAs enriched (positive log fold change) in either DMSO- or selumetinib-treated conditions reflect genetic perturbations that lead to increased growth upon target gene repression while genes with sgRNAs depleted (negative log fold change) reflect genetic perturbations that lead to decreased cell growth upon target gene repression. We first compared DMSO-treated cells and T0 cells to identify genes required for glioblastoma cell growth. In SB28 cells, a total of 1,445 sgRNAs led to significantly decreased cell growth while 137 sgRNAs led to increased cell growth, with *Cdkn2a* and *Tp53* repression comprising 2 of the top enriched sgRNA hits, thus identifying a requirement of *Cdkn2a* or *Tp53* for cell growth, consistent with human tumor mutation data (Supplemental Figure 14A). In GBM43 cells, a total of 690 sgRNAs led to significantly decreased cell growth while 91 sgRNAs led to increased cell growth (Supplemental Figure 14B), and gene ontology analysis of genes required for cell growth in either cell line converged on regulators of cell cycle progression (Supplemental Figure 14, C and D). By integrating genes required for growth in both screens, a 31-gene consensus cell cycle signature required for *NF1*-mutant glioblastoma cell growth was identified that was enriched for components within the cyclin-dependent kinase (CDK), retinoblastoma, or TP53 pathways (Supplemental Figure 14E), consistent with comutations in cell cycle regulatory genes, such as *CDKN2A/B* observed in human tumors (Figure 1A), further serving as a technical validation of our screen in identifying cell cycle genes required for tumor cell growth.

We next evaluated genetic perturbations selectively altering selumetinib response by comparing sgRNA abundance between cells treated with selumetinib compared with cells treated with vehicle DMSO. In SB28 cells, a total of 166 sgRNAs mediated sensitivity and 719 sgRNAs mediated resistance, while in GBM43 cells, 198 sgRNAs mediated sensitivity and 850 sgRNAs mediated resistance (Supplemental Figure 14, F and G). Integration of conserved hits across both screens identified a total of 3 sgRNAs mediating sensitivity and 165 sgRNAs mediating resistance (Figure 4B). We focused on genes mediating selumetinib sensitivity as potentially druggable dependencies, and the top 2 conserved sensitivity hits were *BRAF*, a direct effector of Ras with established roles in glioma (16, 17, 37), and *SHOC2*, which has not been previously implicated in glioblastoma to our knowledge (Figure 4C). SHOC2 functions as part of a heterotrimeric complex whose primary role is to remove an inhibitory phosphorylation on RAF and thus promote RAF activation of MEK (38, 39). Given its role as what we believe to be a novel therapeutic target in glioblastoma, we next validated SHOC2 as a mediator of selumetinib response. CRISPRi *Shoc2* repression in SB28 cells led to decreased cell growth and increased biochemical sensitivity to selumetinib (Figure 4, D and E, and Supplemental Figure 15, A and B). Although we were unable to obtain stable CRISPRi sg*SHOC2* GBM43 cells, siRNA *SHOC2*-deficient GBM43 cells exhibited increased biochemical sensitivity to selumetinib (Figure 4F and Supplemental Figure 15C). We next tested the effect of *Shoc2* repression and selumetinib treatment given at a dose of 25 mg/kg twice daily in SB28 intracranial tumors, demonstrating that *Shoc2* loss combined with selumetinib resulted in more durable responses compared with either perturbation alone (Figure 4G and Supplemental Figure 16, A and B). Taken together, genome-wide CRISPRi screens suggest cell cycle dysregulation is essential for *NF1*-mutant glioblastoma growth and compensatory Ras activation underlies MEK inhibitor responses, with *SHOC2* constituting a potential additional therapeutic target to maximally block Ras pathway output and enhance selumetinib responses in glioblastoma.

## Discussion

*NF1* is recurrently mutated in glioblastoma, yet the availability and efficacy of targeted therapies leveraging this genetic alteration are limited. Here, we combine multiplatform bulk and single-cell genomic analysis of somatic *NF1*-mutant, IDH wild-type glioblastomas with genome-wide CRISPRi screens and mouse intracranial tumor models to better understand the molecular landscape and MEK inhibitor responses in these tumors. Targeted DNA sequencing revealed *EGFR* alteration was mutually exclusive with *NF1* mutation, as previously reported (1–3), and showed an association of *CDKN2A/B* homozygous deletion with worse OS in *NF1*-mutant, but not *NF1* wild-type, glioblastoma. SnRNA-Seq of human *NF1*-mutant glioblastomas revealed multiple transcriptional tumor cell states within and between patients, linking MES-like cell states to Ras/RAF/MEK activation signatures. ScRNA-Seq of vehicle- or selumetinib-treated mouse intracranial tumors demonstrated differences in MEK activation, MEK inhibitor sensitivity, and transcriptional programs underlying MEK inhibitor resistance between MES-like and non-MES tumor cell subpopulations. Genome-wide CRISPRi screens demonstrated additional perturbations in cell cycle genes are required for cell growth while selumetinib sensitivity is dependent on additional Ras pathway outputs, identifying *Shoc2* loss as sufficient to improve selumetinib responses in mouse intracranial glioblastoma tumors. More broadly, it appears the MES-like transcriptional signature classically associated with *NF1* mutation can be effectively treated with maximal Ras/RAF/MEK/ERK pathway blockade while non-MES cells leverage divergent cell cycle and glial differentiation mechanisms to resist Ras/RAF/MEK/ERK pathway blockade. Taken together, our data support a model in which heterogeneity underlies clinical outcomes and MEK inhibitor response within *NF1*-mutant glioblastomas.

The efficacy of molecular monotherapy for malignant tumors such as glioblastoma is limited by numerous factors, including tumor heterogeneity, compensatory intracellular signaling changes, and cellular plasticity, motivating approaches to understand, and ultimately circumvent, treatment resistance. Our observations regarding MEK activation in MES-like cells from *NF1*-mutant glioblastomas may provide the initial steps in defining a molecular biomarker for MEK inhibitor response in *NF1*-mutant glioblastoma. More broadly, devising better approaches to fully block Ras pathway outputs such as through *SHOC2* perturbation or combined pan-RAF (37) plus MEK inhibition may show benefit in depleting the subpopulation of MES-like tumor cells and improving clinical responses to targeted therapy, although further validation of *SHOC2* across additional models will be critical. Although our study focused on *NF1* loss leading to Ras activation, RTKs, such as *PDGFRA* and *EGFR*, which are recurrently mutated in glioblastoma, can also activate Ras signaling through Ras GEFs such as *SOS1*. SHOC2 perturbation to block downstream Ras outputs may thus also be useful in *NF1* intact tumors as observed in other systems (40, 41), and further work is needed to formally test this possibility in glioblastoma. From a translational perspective, while no clinical compounds blocking SHOC2 exist to date, the recent structural elucidation of the SHOC2 heterotrimeric complex has facilitated potential pharmacologic strategies that may be useful in glioblastoma (42–44). However, given *SHOC2* repression did not lead to a sustained treatment response likely due to both compensatory upstream activation of RTK/Ras signaling and tumor heterogeneity within non-MES cells not dependent on Ras signaling, synergistic combinations targeting the parallel susceptibilities of non-MES cells will also be critical, and it will be important to define whether CDK4/6 inhibitors targeting *CDKN2A/B* loss may be useful in this context. The observed intertumor heterogeneity at the level of DNA alterations and intratumor heterogeneity observed in mouse and human scRNA-Seq data (7, 45) underscore the importance of patient selection within *NF1*-mutant glioblastomas. In that regard, the identification of a functional genomic selumetinib sensitivity signature based on our CRISPRi screens may provide a useful tool to identify transcriptional subpopulations, and the patients with tumors that harbor them, in which Ras pathway blockade will prove effective.

In sum, our data support the existence of clinically significant intertumor and intratumor heterogeneity within *NF1*-mutant, IDH wild-type glioblastoma with distinct responses to the MEK inhibitor selumetinib. Future work leveraging larger, multi-institutional, ideally prospective, cohorts will be critical to validate the observation that *CDKN2A/B* loss is a negative prognostic marker and the relationship between comutations and transcriptional subtype. Similarly, the relationship between putative glioblastoma mutational group, single-cell composition, and transitions between primary and recurrent tumors following different therapies will require further investigation to define *NF1*-specific cellular mechanisms underlying targeted therapy resistance, and analysis of matched primary/recurrent pairs will be critical to understand the relationship between treatment selection and tumor evolution. Finally, future preclinical studies and clinical trials aimed at complementing MEK inhibitors with alternative modalities, such as radiation, immunotherapy, or targeted agents against comutated genes such as *CDKN2A/B*, will be critical to achieve durable responses in *NF1*-mutant glioblastomas.

## Methods

### Sex as a biological variable.

Clinical samples representing both sexes were analyzed within the patient cohort in the present study. Female C57BL/6 mice were used as host mice for mouse intracranial allografts in concordance with institutional practice and prior allograft experiments, and we expect these findings will be conserved across male and female host mice.

### Clinical database design and DNA sequencing of human glioblastomas.

Patients treated with surgical resection for a glioblastoma at the UCSF in 2006–2024 were retrospectively identified from a tissue biorepository. Male and female patients 18 years of age or older were included. A total of 186 newly diagnosed IDH wild-type glioblastoma cases containing a pathogenic or likely pathogenic somatic *NF1* mutation using a clinical Clinical Laboratory Improvement Amendments–certified (CLIA-certified) targeted DNA next-generation sequencing assay, obtained as part of routine clinical care, were retrospectively identified. Cases were verified to meet histologic and molecular diagnostic criteria for IDH wild-type glioblastoma as specified in the 2021 WHO Classification of Tumors of the Central Nervous System. Exclusion criteria included any patients with a clinical history or diagnosis of NF-1, a germline *NF1* mutation, or tumors that matched to HGAP using v12.8 of the Heidelberg DNA methylation classifier. Demographic and clinical information was extracted from the electronic medical record and an institutional cancer registry. Survival status was obtained by searching the electronic medical record, institutional cancer registry, Social Security, Department of Motor Vehicles, nationwide hospital databases, and publicly available obituaries. A summary of demographic information is included in Table 1.

Copy number profiles were annotated from a clinical CLIA-certified targeted DNA next-generation sequencing assay. In brief, this capture-based next-generation DNA-sequencing assay contains targets for DNA segments at regular intervals along each chromosome to enable genome-wide copy number and zygosity analyses, with a total sequencing footprint of 2.8 Mb. Genome-wide copy number and zygosity analysis was performed by CNVkit and visualized using NxClinical (Biodiscovery, v6.0). Aggregate genome-wide plots were generated using the R package karyoplotR with the *x* axis representing either whole arm loss or partial loss (proximal or distal) and *y* axis representing percentage of cases containing a specific copy gain or loss.

### Human glioblastoma snRNA-Seq.

Single nuclei were isolated from frozen human sporadic *NF1*-mutant, IDH wild-type glioblastoma surgical resection samples with greater than 30% tumor content using an automated tissue dissociation system (Singulator S100, S2 Genomics). Briefly, 10–20 μg of frozen tumor-rich sample was homogenized and purified using a nuclei isolation kit, buffer containing RNase, and an NIC^+^ cartridge. The resulting suspension was purified using a 40 μm filter and a Percoll gradient. Nuclei were counted using an automated cell counter as well as DAPI staining. Single-nucleus suspensions were processed for snRAN-Seq using the Chromium Single Cell 3’ GEM, Library & Gel Bead Kit v3.1 (1000121, 10x Genomics), and a 10x Genomics Chromium X controller, with cell suspensions diluted per the manufacturer-recommended protocol and settings for a target recovery of 8,000 cells per sample. Libraries were sequenced on an Illumina NovaSeq 6000, targeting more than 50,000 reads per cell, at the UCSF Center for Advanced Technology. Library demultiplexing, read alignment, identification of empty droplets, and unique molecular identifier (UMI) quantification were performed using CellRanger (10x Genomics).

All downstream analyses were performed with Seurat v4.3.0 (46). CellRanger-generated filtered feature matrices were imported into a Seurat object. For quality control, data were filtered on a per-sample basis to remove outliers in gene count, UMI count, mitochondrial genes, and ribosomal genes. The individual count matrices were normalized by SCTransform v2. Scanorama (https://github.com/brianhie/scanorama) (47) was used to perform data integration across datasets, cluster number optimization was performed by comparing multiple cluster resolutions using Clustree, and the selected cluster resolution was examined by silhouette width analysis, which reported a mean width per cluster larger than 0. Genes differentially expressed in each cluster were identified using FindAllMarkers function (cutoff: min.pct = 0.25, logfc.threshold = 0.25, min.diff.pct = 0.2). Tumor versus nontumor cell designation was performed through a combination of manual marker gene inspection and inferCNV (https://github.com/broadinstitute/inferCNV) (48) of the CD45-negative clusters using endothelial cells as reference. Then, using normal cells as reference, SCEVAN (49) (https://github.com/AntonioDeFalco/SCEVAN) was used for identifying tumor subclones based on each individual sample’s CNAs. Cell type designation of nontumor cells was performed through scType automated cell type classification and validated through manual marker gene inspection (50). The automated cell type classification algorithms were developed for normal cells with clearly defined marker genes and become limited when applied to tumor (49) cell types because of the ambiguity of gene signatures of malignant cell types. Therefore, alternative methods were explored to define the different cell states of tumor cells. Tumor cells were assigned to 1 of the 4 meta-modules of AC-like, MES-like, NPC-like, and OPC-like using the cell-scoring methods Neftel et al. established (7). Cycling tumor cells were also defined based on the cell cycle meta-modules signatures by Neftel et al. (7). GSEA was performed using fgsea (v1.27.1) and msigdbr (v7.5.1). For the bubble plots, we showed the mean log2fold change for significant pathways (padj < 0.05).

### Published glioblastoma snRNA-Seq data analysis.

To more broadly understand how NF1 mutation or CDKN2A/B deletion affects cellular populations, we obtained a published glioblastoma snRNA-Seq dataset from Wang et al. (28, 29). From this dataset, a total of 53 patient samples with UCSF 500 Cancer Gene Panel Test genotyping validation were analyzed. Cells with less than 5% mitochondrial read counts and at least 200 expressed genes were retained for analysis. Then, UMAP clustering and tumor/nontumor cell annotation were performed the same way as previously published (28, 29). Cell type designation of nontumor cells was validated through manual marker gene inspection (50). Tumor cells were assigned to 1 of the 4 meta-modules of AC-like, MES-like, NPC-like, and OPC-like using the cell-scoring methods Neftel et al. established (7). Cycling tumor cells were also defined based on the cell cycle meta-modules signatures by Neftel et al. (7). GSEA was performed using fgsea (v1.27.1) and msigdbr (v7.5.1). For the bubble plots, we showed the mean log2fold change for significant pathways (padj < 0.05).

### Mouse intracranial tumor establishment, bioluminescence imaging, and drug treatment.

Female 5- to 6-week-old C57BL/6 mice (Envigo Laboratories), housed under aseptic conditions, received intracranial tumor cell injection through the UCSF Brain Tumor Center Preclinical Therapeutics Core. Briefly, mice were anesthetized by combination of intraperitoneal injection of a mixture containing ketamine (100 mg/kg) and xylazine (10 mg/kg) and of inhalation of isoflurane. The scalp was surgically prepped, and a skin incision ~10 mm in length was made over the middle frontal to parietal bone. The surface of the skull was exposed so that a small hole could be made 3.0 mm to the right of the bregma and just in front of the coronal suture with a 25-gauge needle. A 26-gauge needle attached to a Hamilton syringe was inserted into the hole in the skull. The needle was covered with a sleeve that limits the injection depth to 3–4 mm. A total of 3 μL of tumor cell suspension was injected into the right caudate putamen at a rate of 1 μL/min by free hand. The skull surface was then swabbed with hydrogen peroxide before the hole was sealed with bone wax to prevent reflux. The scalp was closed with surgical staple. Mice were treated with either selumetinib 25 mg/kg or with vehicle control by oral gavage twice daily. For bioluminescence imaging, mice were anesthetized with inhalation of isoflurane, then administered 150 mg/kg of luciferin (d-luciferin potassium salt, Gold Biotechnology) via intraperitoneal injection. Ten minutes after luciferin injection, mice were examined for tumor bioluminescence with an IVIS Lumina imaging station and Living Image software (Caliper Life Sciences), and intracranial regions of interest were recorded as photons per second per steradian per square centimeter.

### Mouse intracranial allograft scRNA-Seq.

Harvested tumors were mechanically minced with forceps and then dissociated to single-cell suspension using the Papain Dissociation System (Worthington LK003150) following the manufacturer’s protocol. To further remove noncellular debris, cell suspensions were passed through a 70 μM strainer (Corning, 352350), centrifuged at 300*g* for 5 minutes, and resuspended in cold phosphate-buffered saline.

Similar to human snRNA-Seq, intracranial allograft single-cell sequencing was performed using the Chromium Single Cell 3’ Library & Gel Bead Kit v3.1 on a Chromium controller (10x Genomics) using the manufacturer-recommended default protocol and settings. Samples were sequenced on an Illumina NovaSeq 500 at the UCSF Center for Advanced Technology, and the demultiplexed FASTQ files were processed using CellRanger for alignment to the mm10 reference genome, identification of empty droplets, and determination of a count threshold. All downstream analyses were performed with Seurat v4.4 (46). CellRanger-generated filtered feature matrices were imported into a Seurat object. For quality control, data were filtered on a per-sample basis to remove outliers in gene count, UMI count, mitochondrial genes, and ribosomal genes. The individual count matrices were normalized by SCTransform v2. Scanorama was used to perform data integration across datasets, cluster number optimization was performed by comparing multiple cluster resolutions using Clustree, and the selected cluster number was verified with silhouette scores. Genes differentially expressed in each cluster were identified using FindAllMarkers function (cutoff: min.pct = 0.25, logfc.threshold = 0.25, min.diff.pct = 0.1). Tumor versus nontumor cell designation was based on gene expression but not CNAs because the algorithms available for inferring CNAs were developed for human cells only. Using the normal cells as reference, SCEVAN (49) (https://github.com/AntonioDeFalco/SCEVAN) was used for identifying tumor subclones based on each individual sample’s CNAs. Nontumor cells were identified by a combination of CD45 expression, manual marker gene inspection, and scType automated cell type classification. Tumor cells were assigned to 1 of the 4 meta-modules of AC-like, MES-like, NPC-like, and OPC-like using the cell-scoring methods by Neftel et al. (7). Cycling tumor cells were also defined based on the cell cycle meta-modules signatures by Neftel et al. (7). Genes differentially expressed in each cluster in selumetinib-treated compared with vehicle-treated samples were identified using FindMarkers function (cutoff: min.pct=0.2, logfc.threshold=0), and the results were plotted using EnhancedVolcano (cutoff: logFCcutoff=0, pvalCutoff=0.05). GSEA was performed using fgsea (v1.27.1) and msigdbr (v7.5.1). For the bubble plots, we showed the mean log2fold change for significant pathways (padj < 0.05).

### Human GBM cell line scRNA-Seq.

The GBM43 *NF1*-mutant glioblastoma model was a gift from Jann Sakaria and the Mayo Clinic Brain Tumor Patient-Derived Xenograft National Resource (Rochester, Minnesota, USA). GBM43 cells were treated for 18 hours with vehicle, selumetinib, or trametinib. Multi-seq hashing was performed to eliminate batch effects, followed by scRNA-Seq with the Chromium Single Cell 3’ Library & Gel Bead Kit v3.1 on a Chromium controller using the manufacturer-recommended default protocol and settings. Samples were sequenced on an Illumina NovaSeq 500 at the UCSF Center for Advanced Technology, and the demultiplexed FASTQ files were processed using CellRanger. All downstream analyses were performed with Seurat v4.4 (46). Tumor cells were analyzed using the methods established by Neftel et al. (7).

### Tissue culture.

HEK293T cells obtained from the McCormick Laboratory at UCSF were cultured in DMEM (Gibco, 11960069) supplemented with 10% FBS (Life Technologies, 16141) and 1× Pen-Strep (15140122, Life Technologies). SB28 mouse glioblastoma cells were cultured in RPMI with 10% FBS and 1% HEPES, sodium pyruvate, nonessential amino acids, Pen-Strep, Glutamax, and β-mercaptoethanol. GBM43 cells were cultured as neurospheres in N5 media without serum. Cell cultures were obtained from institutional investigators and authenticated by short tandem repeat analysis at the UC Berkeley DNA Sequencing Facility as well as routinely tested for mycoplasma using the MycoAlert Detection Kit (Lonza, 75866-212) as part of standard practice.

For siRNA experiments, cells were transfected with jetOPTIMUS transfection reagent and buffer (Polyplus Transfection) using siRNA against *SHOC2*. Transfections were carried out according to jetOPTIMUS kit instructions for reverse transfection. Briefly, siRNAs were combined with jetOPTIMUS transfection buffer and reagent with a 1:1 ratio of nucleic acid/reagent and incubated at room temperature for 10 minutes. This master mix was then added to a 6-well plate prepared with normal N5 media. Cells were then split normally and added to the 6-well plate with 400,000 cells per well. Cells were then incubated as normal, and siRNA knockdown of *SHOC2* was observed for 24–48 hours following transfection.

### Immunoblotting.

Whole-cell lysates were harvested using standard methods in RIPA buffer (50 mM Tris-HCl at pH 8.0, 150 mM NaCl, 0.5% deoxycholate, 0.1% SDS, 1% IGEPAL CA630) with fresh protease (P8340, Sigma) and phosphatase inhibitor (P2850, Sigma) cocktails. A total of 15 μg of protein was loaded into precast NuPAGE electrophoresis gels (Life Technologies). Samples were separated by SDS-PAGE, transferred to nitrocellulose or PVDF membranes, and blocked in either 5% bovine serum albumin or 5% skim milk in TBS buffer for 1 hour at room temperature. Primary antibodies were incubated overnight at the indicated dilutions at 4°C, and HRP-conjugated secondary antibodies were incubated for 1 hour at room temperature followed by ECL-based detection on film. The following primary antibodies were used: phospho-ERK (Cell Signaling Technologies, 4370, 1:1,000 dilution), total ERK (Cell Signaling Technologies, 4695, 1:1,000 dilution), phospho-MEK (Cell Signaling Technologies, 9121, 1:1,000 dilution), total MEK (Cell Signaling Technologies, 8727, 1:1,000 dilution), and SHOC2 (Cell Signaling Technologies, 53600, 1:1,000 dilution). Secondary antibodies used were Goat anti-rabbit IgG (H + L)-HRP Conjugate (BioRad, 1706515, 1:1,000 dilution) and Goat Anti-Mouse IgG (H + L)-HRP Conjugate (BioRad, 1706516, 1:1,000 dilution).

### CRISPRi cell line generation and genome-wide screening.

Lentivirus containing pMH0001 (UCOE-SFFV-dCas9-BFP-KRAB, 85969, Addgene) was produced from transfected HEK293T cells with packaging vectors (pMD2.G 12259, Addgene, and pCMV-dR8.91, Trono Lab) following the manufacturer’s protocol (MIR6605, Mirus). SB28 and GBM43 cells were stably transduced to generate parental dCas9-KRAB-BFP cells and selected by flow cytometry using an SH800 sorter (Sony). Subsequent gene-specific knockdowns were achieved by individually subcloning sgRNA protospacer sequences into the pCRISPRia-v2 vector (84832, Addgene) between BstXI and BlpI restriction sites. All constructs were validated by Sanger sequencing of the protospacer region. The following protospacers were used: sgNTC (GTGCACCCGGCTAGGACCGG), sghSHOC2-1 (GGGCAGCGTCGCTTCTTAGG), sghSHOC2-2 (GGGCTCCTGACGGTAACTCG).

For human GBM43 genome-wide CRISPRi screening, we used a compact and highly active sgRNA library containing the top 2 on-target sgRNAs for 23,483 genes that was optimized through aggregation of 126 genome-wide CRISPRi screens, established sgRNAs targeting essential genes, and machine learning prediction algorithms (51). This genome-wide dual sgRNA library has been previously validated through multiple growth-based screens as well as through confirmation of on-target gene repression using perturb-seq, exhibiting 82%–92% median target knockdown. The genome-wide dual sgRNA library was cloned into the library expression vector pU6-sgRNA Ef1alpha Puro-T2A-GFP derived from pJR85 (140095, Addgene) and modified to express a second sgRNA using the human U6 promoter as previously described. We also included 1,137 nontargeting sgRNA pairs as negative controls in the screen. To generate lentiviral pools, we transfected HEK293T cells with the sgRNA library along with packaging plasmids as described above, and viral supernatant was collected 72 hours following transfection. Three replicates of each screen were performed at a coverage of 1,000× cells per sgRNA. Lentiviral libraries were transduced into GBM43 dCas9-KRAB-BFP cells at an MOI of ~0.1, cultured for 2 days following infection, selected in 0.5 μg/mL puromycin for 2 days, and then allowed to recover in standard growth medium for 1 day. Infection efficiency was evaluated by measuring GFP positivity on flow cytometry, and cell pellets were subsequently frozen down at this T0 time point. Cells were cultured in either 1 μM selumetinib or vehicle (DMSO) control for 16 days, which correlated with ~5 population doublings in the selumetinib condition. Cell pellets were frozen down at this T16 time point for both vehicle- and selumetinib-treated conditions. Samples were then processed for sgRNA abundance library preparation using Q5 High-Fidelity DNA Polymerase (New England Biolabs) and sequenced on an Illumina NextSeq 500 (52). Downstream analysis was carried out as previously described (20). In brief, enrichment or depletion of sgRNA abundances was determined by downsampling trimmed sequencing reads to equivalent amounts across all samples, then calculating the log2 ratio of sgRNA abundance in experimental conditions to sgRNA abundance in vehicle conditions at T16, or between sequencing reads from T16 and T0 time points within experimental or control conditions. Specifically, we computed normalized log2 ratios for selumetinib-treated sgRNA abundance at T16 compared with vehicle-treated sgRNA abundance at T16 to identify mediators of selumetinib responses. Statistical significance was calculated using Wald’s test comparing replicates across conditions without a log2 fold change threshold. Hits were prioritized by normalizing log2 ratios to the total number of population doublings in the screen and the standard deviations of the nontargeting control sgRNAs. These phenotype log2 ratios were used for subsequent analysis and visualization. Genes were filtered at an adjusted *P* < 0.05 for further analysis.

For mouse SB28 genome-wide CRISPRi screening, we used a genome-wide mouse sgRNA library targeting 20,003 genes at 5 sgRNAs/gene, for a total of 107,415 sgRNAs, in addition to 2,170 nontargeting control sgRNAs (53). Pooled lentivirus was generated as above, and SB28 cells were transduced with the addition of polybrene (8 μg/mL) at an MOI of 0.1 and were confirmed via flow cytometry 48 hours following lentivirus transduction. One day of puromycin selection (2.0 μg/mL) was performed, followed by 1 day of growth in nonpuromycin 10% FBS in DMEM. Two replicates of each screen were performed at a coverage of 1,000× cells per sgRNA, in both vehicle and 1 μM selumetinib conditions. Infection efficiency was evaluated by measuring GFP positivity on flow cytometry. Initial (T0) cell populations were frozen in 10% DMSO and processed for genomic DNA using the NucleoSpin Blood XL Kit (MACHEREY-NAGEL, 740950.50). Endpoint cell pellets were harvested for genomic DNA after 10 days of growth, corresponding to ~10 and ~5 doublings in the vehicle and selumetinib conditions, respectively. sgRNA-sequencing libraries were prepared using NEBNext Ultra II Q5 PCR MasterMix (New England Biolabs, M0544L) and sequenced on an Illumina NextSeq 500.

Growth phenotype (gamma) was defined as log2(sgRNA count vehicle/sgRNA count T0) minus median sgNTC log2(sgRNA count vehicle/sgRNA count T0), then normalized by the number of cell doublings, as previously described (20). Drug phenotype (tau) was defined as log2(sgRNA count selumetinib/sgRNA count T0) minus median sgNTC log2(sgRNA count selumetinib/sgRNA count T0), then normalized by the number of cell doublings in the drug screen. Drug/growth ratio phenotype (rho) was defined as log2(sgRNA count selumetinib/sgRNA count vehicle), then normalized by the number of cell doublings in the drug screen. Gene-level phenotypes were summarized as the mean of the top 3 sgRNAs against a given gene, ranked according to screen phenotype. Statistical significance was calculated using Mann-Whitney *U* test for a given perturbation compared with the sgRNA distribution of the nontargeting control sgRNAs.

### Nucleic acid extraction and quantitative reverse transcription PCR.

From glioblastoma cell lines, RNA was extracted using the RNeasy Mini Kit (74106, QIAGEN) according to manufacturer’s instructions. cDNA was synthesized from RNA using iScript cDNA Synthesis kit (1708891, Bio-Rad). Real-time quantitative PCR was performed using PowerUp SYBR Green Master Mix (A25918, Thermo Fisher Scientific) on a QuantStudio 6 Flex Real Time PCR system (Life Technologies) and analyzed using the ΔΔ method as previously reported (20). The following quantitative PCR primers were used: GAPDH-F (5′-GTCTCCTCTGACTTCAACAGCG-3′), GAPDH-R (5′-ACCACCCTGTTGCTGTAGCCAA-3′), hSHOC2-F (5′-GTTGACAATACGATCAAACGGC-3′), hSHOC2-R (5′-CTCTTCCCGGCATTTGTTGAG-3′), mSHOC2-F (5′-AATACCATCAAACGGCCAAATCC-3′), and mSHOC2-R (5′-AACCGCATTGAGTTCTCCTCC-3′).

### Statistics.

All statistical analyses were performed in GraphPad Prism (v10.0.0) or R (v3.5.3 and v3.6.1). Statistical tests and number of replicates are indicated in figure legends. Kaplan-Meier curves were compared using the log-rank test. χ^2^ tests were used to compare categorical variables. The Wald test was used to assess the gene knockdown effects in CRISPRi screens. The Wilcoxon test was used to compare single-cell gene expression values. When used, 2-tailed Student’s *t* tests were performed for all comparisons. A *P* value of less than 0.05 was considered significant for all comparisons.

### Study approval.

Use of archived human glioblastoma specimens and associated clinical data was approved by the UCSF Institutional Review Board under protocols 10-01318 and 22-37134. All animal procedures were performed under a protocol approved by the UCSF Institutional Animal Care and Use Committee under protocol AN200569-00.

### Data availability.

The data that support the findings of this study are available in the Supporting Data Values file. Human tumor snRNA-Seq (*n* = 9) and intracranial tumor scRNA-Seq (*n* = 6) reported in this manuscript have been deposited in the NCBI Gene Expression Omnibus under BioProject PRJNA111929.

The open-source software, tools, and packages used for data analysis in this study, as well as the version of each program, were ImageJ (NIH) (v2.1.0), R (v3.5.3 and v3.6.1), CellRanger (v6.1.2), Seurat R package (v4.4.0), Clustree (v0.5.0), Scanorama (v1.7.3), minfi (Bioconductor v3.10), ConsensusClusterPlus (Bioconductor v3.10), Heatmap.2 R package (gplots v3.13), and ggplot2 (v3.4.3). No custom software, tools, or packages were used. CRISPRi screen analysis code is available at https://github.com/liujohn/CRISPRi-dual-sgRNA-screens/blob/main/module2/PhenotypeScores.R (commit ID de22928). scRNA-Seq analysis code is available at https://github.com/Vasudevan-Lab/nf1_gbm (commit ID b1d2f68).

## Author contributions

SP, KM, and HNV designed the study. SP, KM, EP, SJL, RG, JW, JP, and TO conducted experiments. JSY, BN, ATR, SEB, JJP, SC, DAS, AP, DRR, and MSB acquired data and coordinated clinical sample/database management. SP, KM, EP, SJL, NAA, ZP, PS, and HNV analyzed data. ARA and HNV supervised the work. SP, KM, and HNV drafted the manuscript. All authors reviewed, edited, and approved the final version of the manuscript.

## Supplementary Material

Supplemental data

Unedited blot and gel images

Supplemental tables 1-12

Supporting data values

## Figures and Tables

**Table 1:**
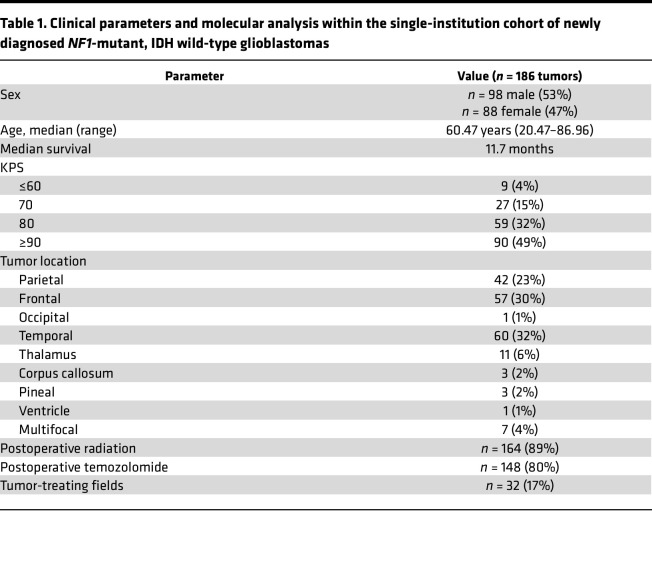
Clinical parameters and molecular analysis within the single-institution cohort of newly diagnosed *NF1-*mutant, IDH wild-type glioblastomas

## Version 1 09/23/2025

Electronic publication
