# Supplementary material for: Tumor heterogeneity underlies clinical outcome and MEK inhibitor response in somatic NF1-mutant glioblastoma
Source: JCI Insight. 2025 Sep 23;10(18):e192658. doi: 10.1172/jci.insight.192658 (PMC12487855; doi:10.1172/jci.insight.192658)
Supplement: Supplemental data [file jciinsight-10-192658-s009.pdf]

Supplementary Figures

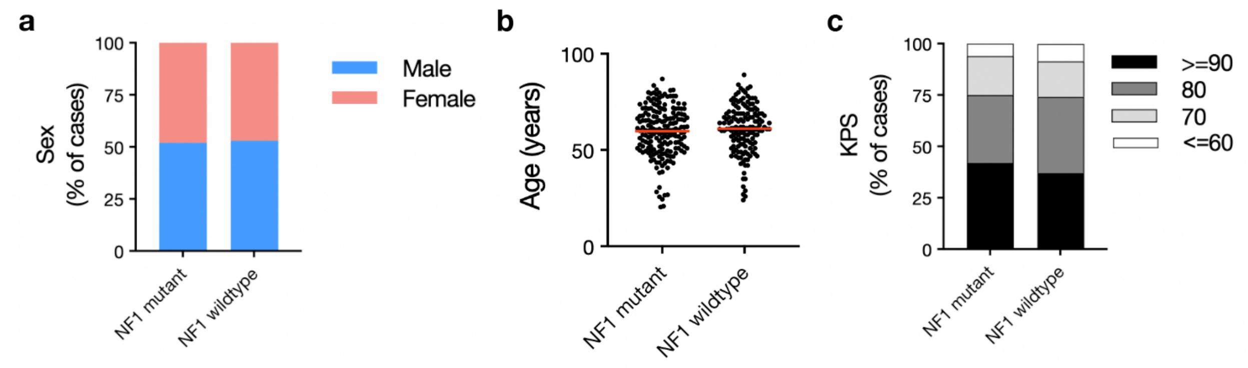

Supplementary Figure 1. Paired analysis of the *NF1* mutant, IDH-wildtype glioblastoma cohort with a propensity score matched *NF1* wildtype, IDH-wildtype glioblastoma cohort. Comparison of baseline clinical parameters between *NF1* mutant and *NF1* wildtype propensity score matched cohorts shows no differences in a. sex, b. age, or c. KPS.

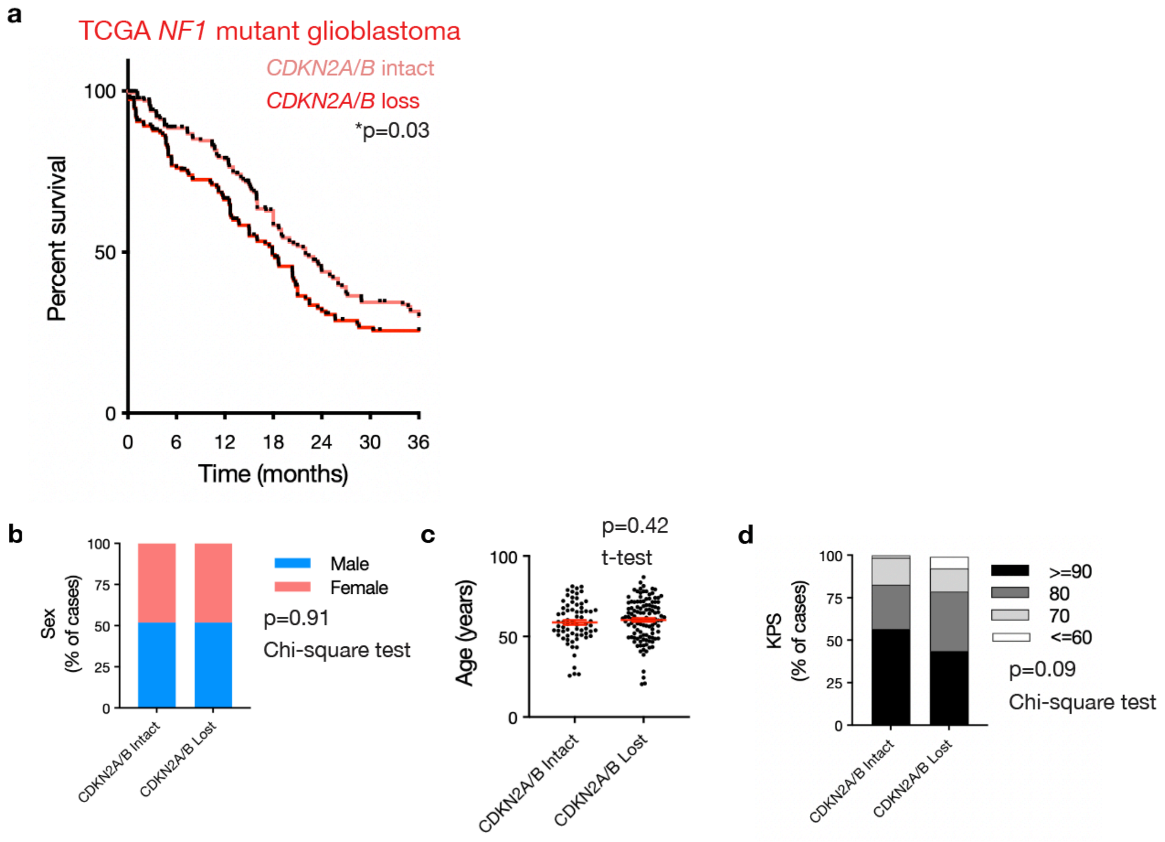

Supplementary Figure 2. *CDKN2A/B* status in *NF1* mutant, IDH-wildtype glioblastomas and relationship to baseline clinical or pathologic variables. a. Analysis of *NF1* mutant, IDH-wildtype glioblastomas from The Cancer Genome Atlas (TCGA) cohort shows *NF1* mutant, *CDKN2A/B*-deleted glioblastomas are associated with worse overall survival compared to *NF1* mutant, *CDKN2A/B*-intact tumors (p=0.03). b. No significant difference was observed in biological sex (p=0.91, Chi-square test), c. age (p=0.42, unpaired t-test) or d. KPS (p=0.09, Chi-Square test) within *NF1* mutant tumors based on *CDKN2A/B* status.

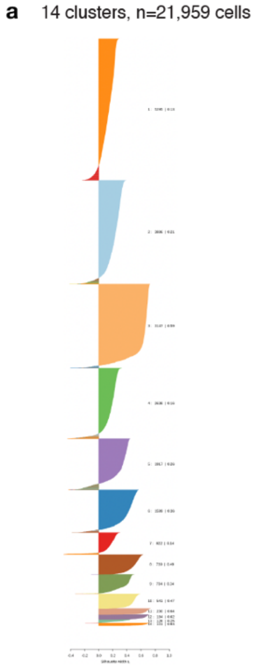

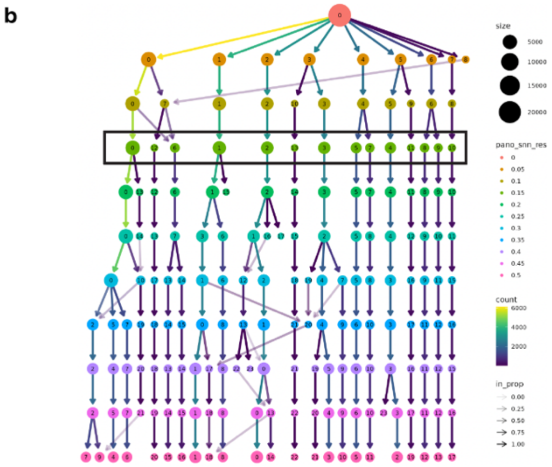

c

Cluster	0	1	2	3	4	5	6	7	8	9	10	11	12	13
S1	1831	1	0	1	1	0	813	0	0	0	0	0	125	0
S2	3444	65	118	5	122	0	3	0	106	98	0	0	1	0
S3	0	52	676	12	224	0	0	0	43	2	0	5	0	2
S4	0	1638	5	4	67	0	0	0	23	9	0	0	0	4
S5	7	1153	1458	8	701	6	0	0	313	242	12	131	0	12
S6	1	612	4	23	267	1	0	0	0	0	0	1	0	54
S7	2	30	659	2514	316	4	0	1	196	177	36	35	0	14
S8	0	291	93	19	96	1583	0	738	0	0	0	5	0	1
S9	10	44	134	52	123	5	6	0	53	13	182	7	0	14

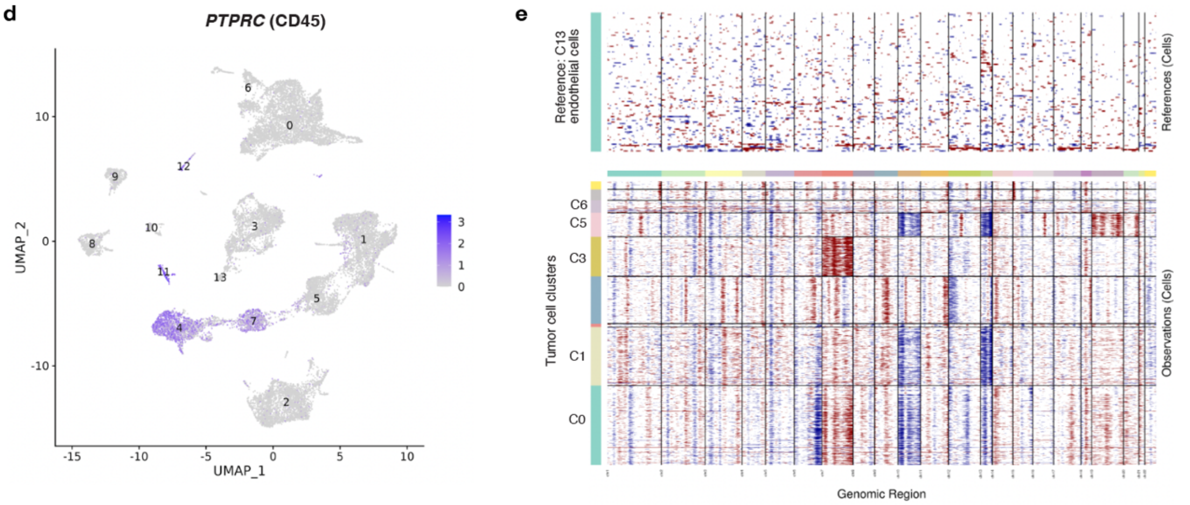

Supplementary Figure 3. Single nuclear RNA-sequencing analysis of human *NF1* mutant, IDH-wildtype glioblastomas (n=9). a. Silhouette analysis and b. cluster tree reveals 14 clusters across 21,959 single nuclei leads to robust cell type classification. c. Summary of number of cells per cluster by sample of origin. d. *PTPRC* (CD45) expression marks non-tumor cells from the hematopoietic lineage. e. inferCNV identifies tumor cell clusters (clusters 0, 1, 3, 5, 6,) containing CNAs in single cell RNA-seq data compared to non-tumor populations with normal relative ploidy.

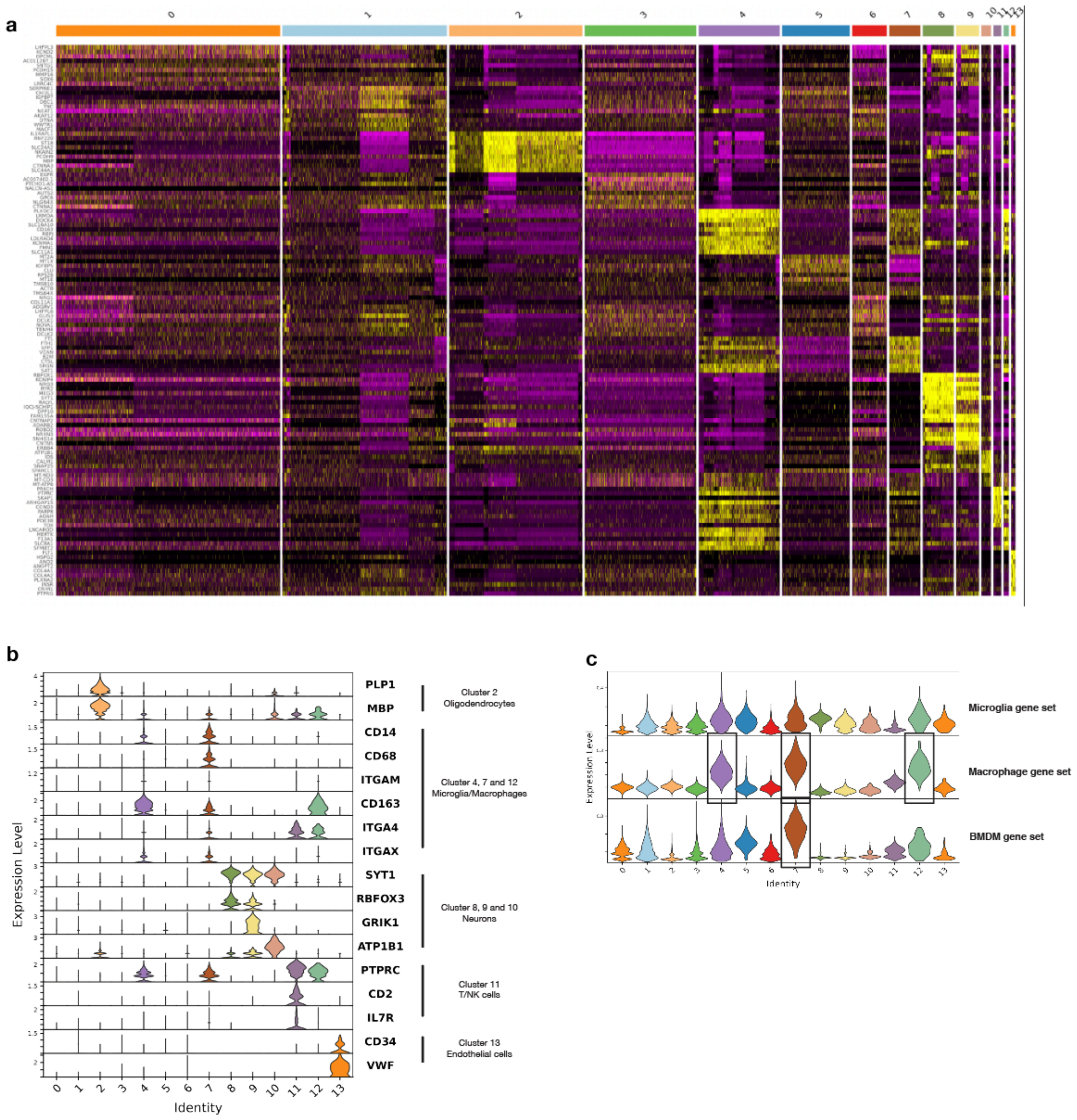

Supplementary Figure 4. Marker gene analysis and cluster assignment from snRNA-sequencing of human *NF1* mutant glioblastomas. a. Heatmap for cluster marker genes across all clusters. b**.** Violin plots of selected marker genes for non-tumor cell types. c. Violin plot of microglia, macrophages, or BMDM gene sets used to define non-tumor cell subpopulations.

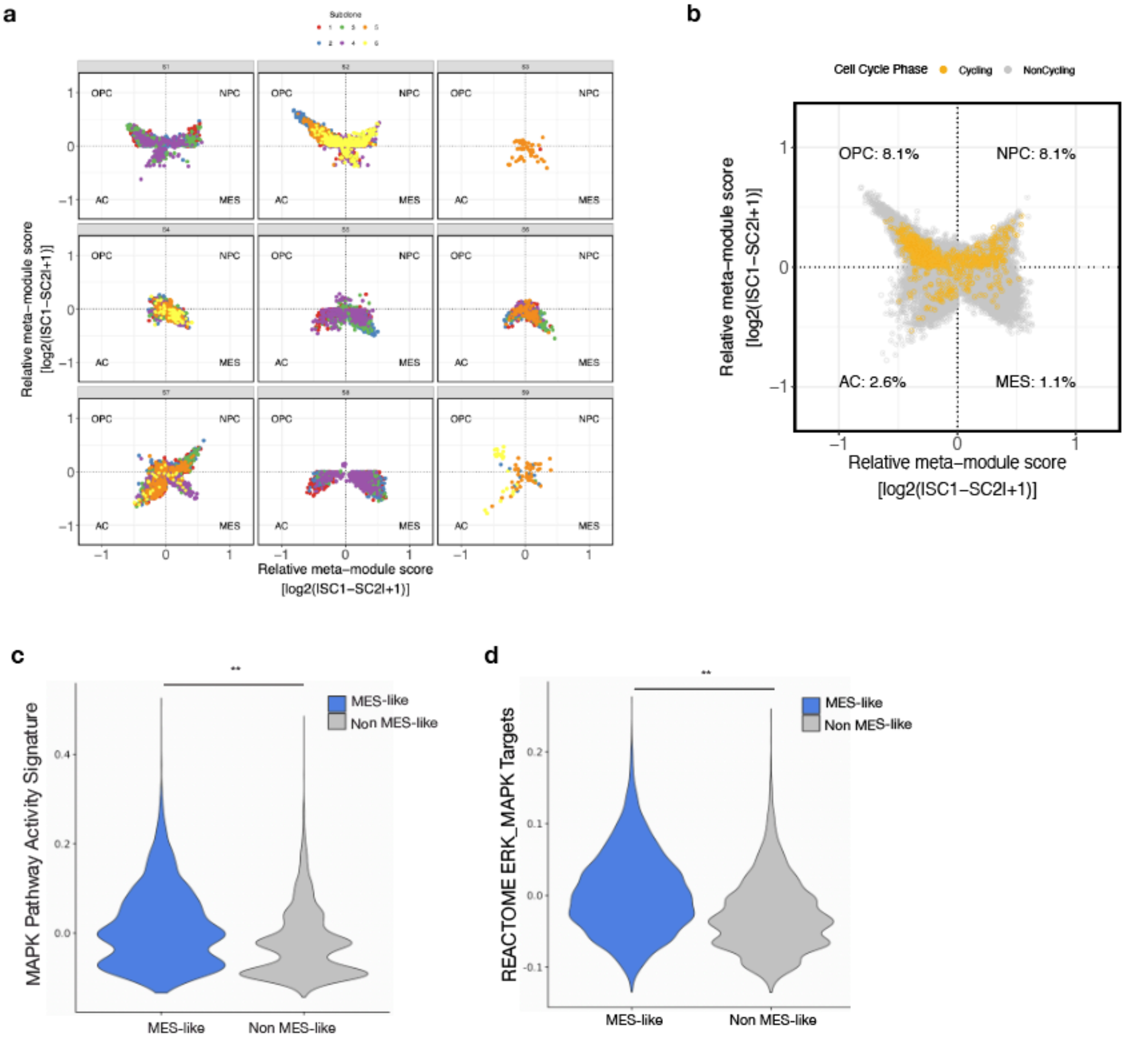

Supplementary Figure 5. Tumor cells from snRNA-sequencing of human *NF1* mutant, *CDKN2A/B* deleted glioblastomas. a. Tumor cell states are distributed across four transcriptional states (OPC-like, NPC-like, AC-like, MES-like) comprising different subclones. b. Cycling cells were overrepresented in the OPC-like state (8.1%) and NPC-like state (8.1%) compared to MES-like or AC-like cells. c. MES-like cells express significantly increased MAPK pathway activity compared to non-MES cells (** $p < 0.001$, Wilcoxon test. d. MES-like cells express significantly increased ERK/MAPK targets compared to non-MES cells (** $p < 0.001$, Wilcoxon test.

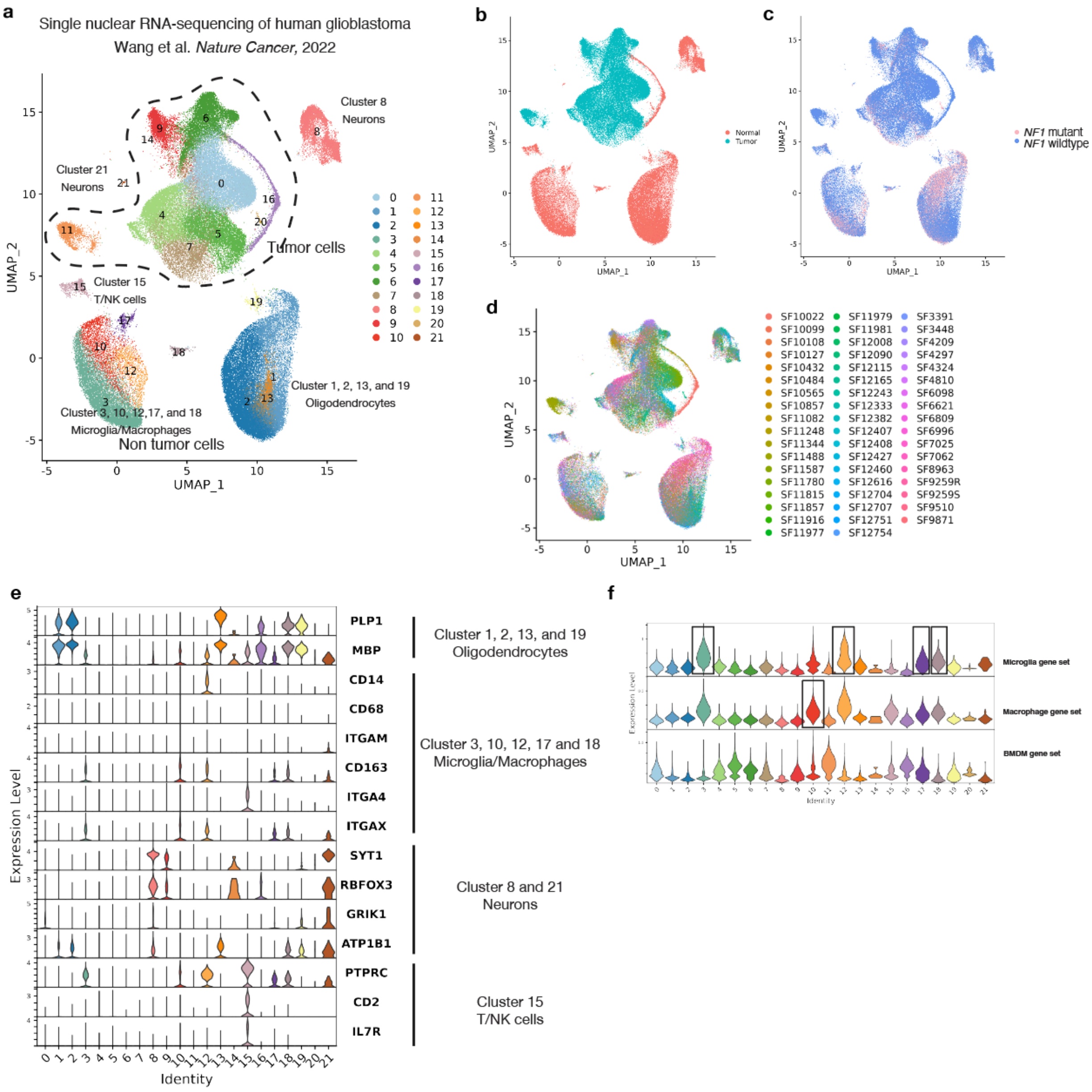

Supplementary Figure 6. Single nuclear RNA-sequencing of human glioblastoma reveals significant overlap between *NF1* wildtype and *NF1* mutant glioblastoma cell clusters. a. Published single nuclear RNA-sequencing (snRNA-seq) of human glioblastomas reveals 21 cell clusters comprising b. 10 tumor cell clusters and 12 non tumor cell clusters that c. predominantly overlapped by *NF1* mutation status and d. by sample of origin. e**.** Violin plots of selected marker genes for non-tumor cell types. f. Violin plot of microglia, macrophages, or BMDM gene sets.

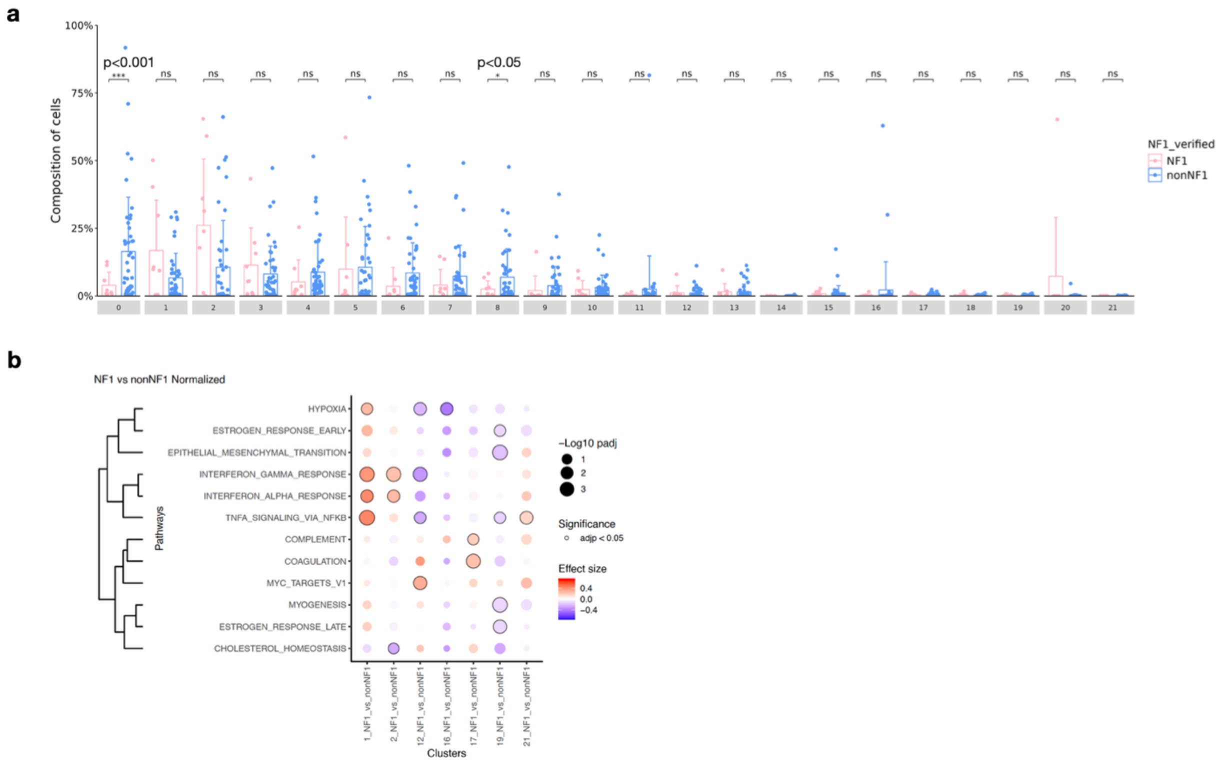

Supplementary Figure 7. *NF1* mutant glioblastomas harbor complex microenvironmental changes compared to *NF1* wildtype glioblastomas. a. Among non-tumor cell populations, *NF1* mutant tumors have significantly fewer neurons (C8) compared to *NF1* wildtype tumors. b. Differential gene expression analysis revealed *NF1* mutant tumors upregulate pro-inflammatory interferon pathways in oligodendrocytes (C1, C2) but downregulate these pathways in microglia/macrophages (C12)

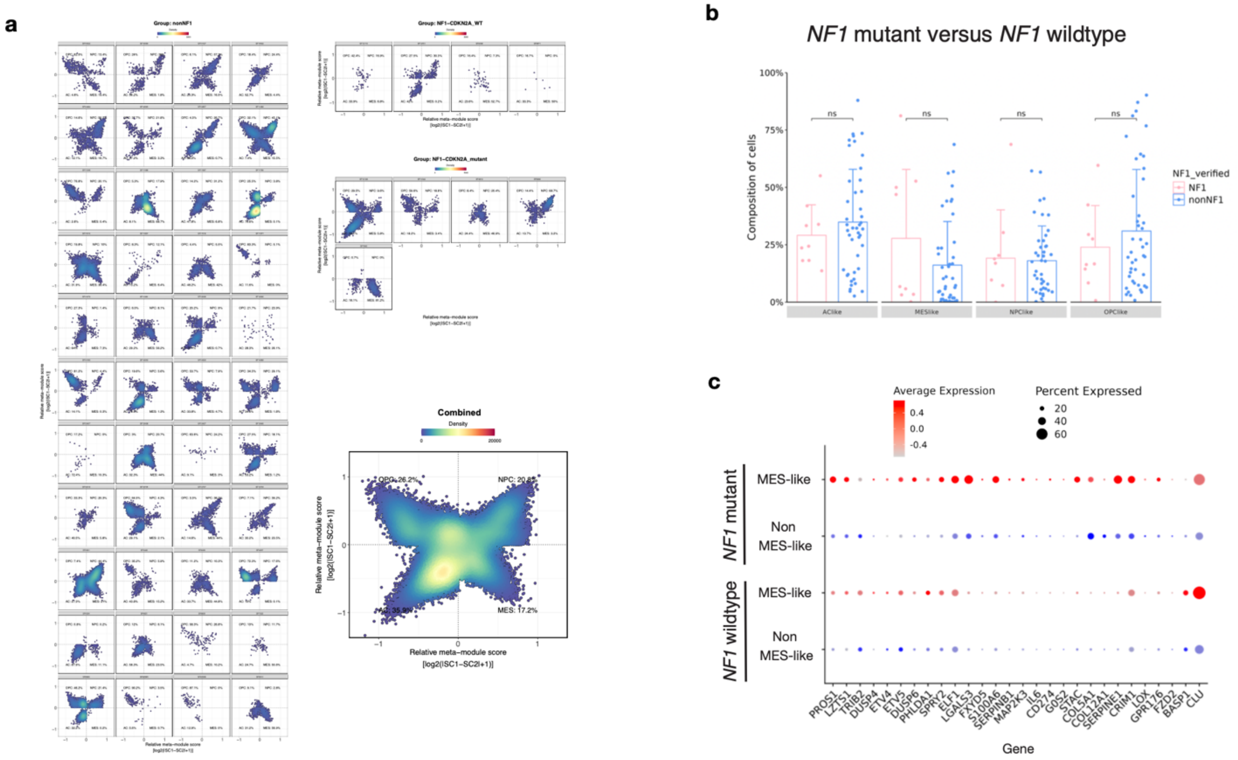

Supplementary Figure 8. *NF1* mutation influence glioblastoma tumor cell states and MEK activation signatures. a. Tumor cells were classified into four states (OPC-like, NPC-like, AC-like, MES-like) and organized based on *NF1* wildtype or *NF1* mutant with or without *CDKN2A/B* deletion. b. No significant differences in cell state proportions were observed between *NF1* mutant and *NF1* wildtype tumors although there was a trend toward increased MES-like cells in *NF1* mutant glioblastomas. c. Single cell dot plot expression analysis of 31 genes comprising the MEK activation gene set reveals enrichment in MES-like cells within *NF1* mutant samples.

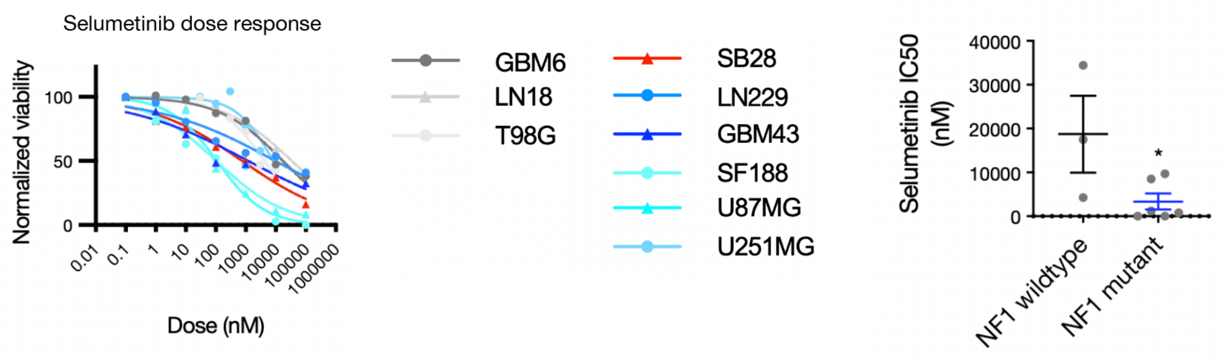

NF1 wildtype

GBM6: *EGFR* amplified
LN18: *PIK3CA*/*CDKN2A*/*TP53* mutant
T98G: *PTEN*/*TP53* mutant

NF1 mutant

LN229
GBM43
SF188
U87MG
U251MG

Mouse SB28
NrasG12V; Tp53 mutant

Supplementary Figure 9. *NF1* mutant glioblastoma cell lines show greater sensitivity to selumetinib compared to *NF1* wildtype cell lines in vitro (*p=0.04, t-test).

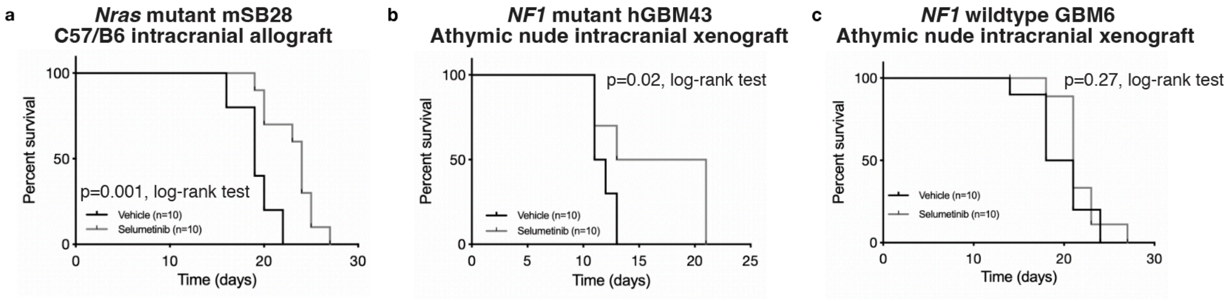

Supplementary Figure 10. Selumetinib significantly prolongs survival in SB28 (NrasG12V mutant) and GBM43 (*NF1* mutant) intracranial glioblastoma models but not in GBM6 (*NF1* wildtype) intracranial glioblastomas.

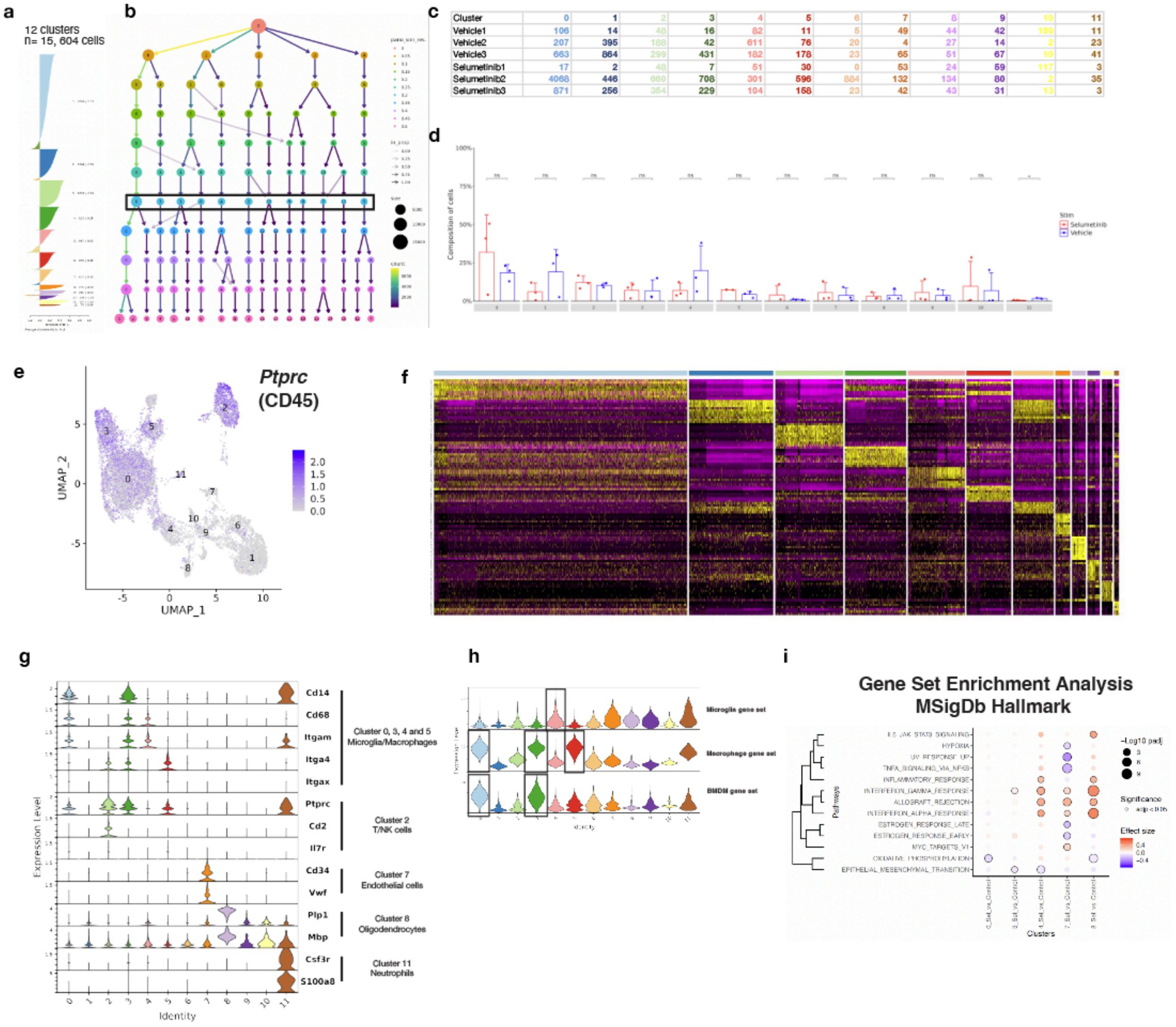

Cluster	0	1	2	3	4	5	6	7	8	9	10	11
Vehicle1	106	14	48	16	82	11	5	49	44	42	109	11
Vehicle2	207	395	188	42	611	76	20	4	27	14	2	23
Vehicle3	663	864	299	431	182	178	23	65	51	67	10	41
Selumetinib1	17	2	48	7	51	30	0	53	24	59	117	3
Selumetinib2	4068	446	660	708	301	596	884	132	134	80	2	35
Selumetinib3	871	256	354	229	104	158	23	42	43	31	13	3

Supplementary Figure 11. Single cell RNA-sequencing of SB28 intracranial allografts reveals 12 clusters comprised of both tumor and non-tumor cells. a. Silhouette analysis and b. cluster tree reveals 12 clusters leads to robust cell type classification. c. Summary of number of cells per cluster by sample of origin. e. *Ptprc* (CD45) expression marks non-tumor cells from the hematopoietic lineage. f. Marker gene expression and g. violin plot of selected marker genes for non-tumor cell types. h. Violin plot of microglia, macrophages, or BMDM gene sets. i. Differential gene expression and gene set enrichment analysis (GSEA) between selumetinib-treated and vehicle-treated conditions reveals MEK inhibition leads to a pro-inflammatory signature in the microenvironment as evidenced by induction of interferon

mediated inflammatory responses in microglia (C4), endothelial cells (C7), and oligodendrocytes (C8).

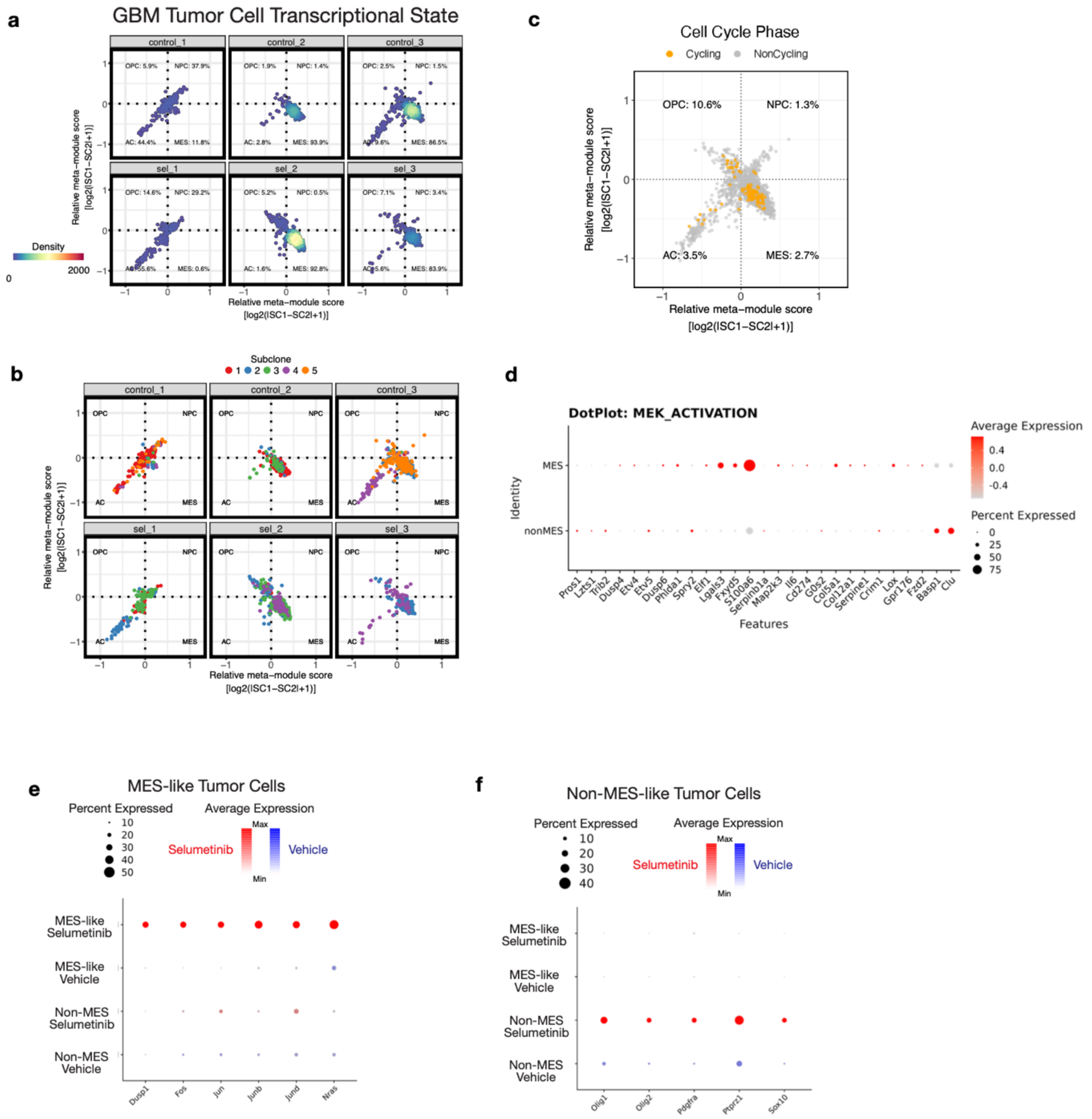

Supplementary Figure 12. Single cell RNA-sequencing analysis of tumor cells from SB28 allografts treated with selumetinib reveals differential mechanisms of MEK inhibitor resistance between MES-like and non-MES tumor cell subpopulations. a. Tumor cell states are distributed across four transcriptional states (OPC-like, NPC-like, AC-like, MES-like) and b. different subclones. c. Cycling cells were overrepresented in the OPC-like states (10.6%). d.

Single cell dot plot expression analysis of 31 genes comprising the MEK activation gene set reveals enrichment in MES-like cells compared to non-MES cells. e. MES-like cells exhibit Ras pathway activation with increased expression of *Fos, Jun,* and *Dusp1* following selumetinib treatment. f. Non-MES cells show induction of glial differentiation genes (*Olig1, Sox10, Pdgfra*) with selumetinib treatment, suggesting distinct mechanisms of response and resistance.

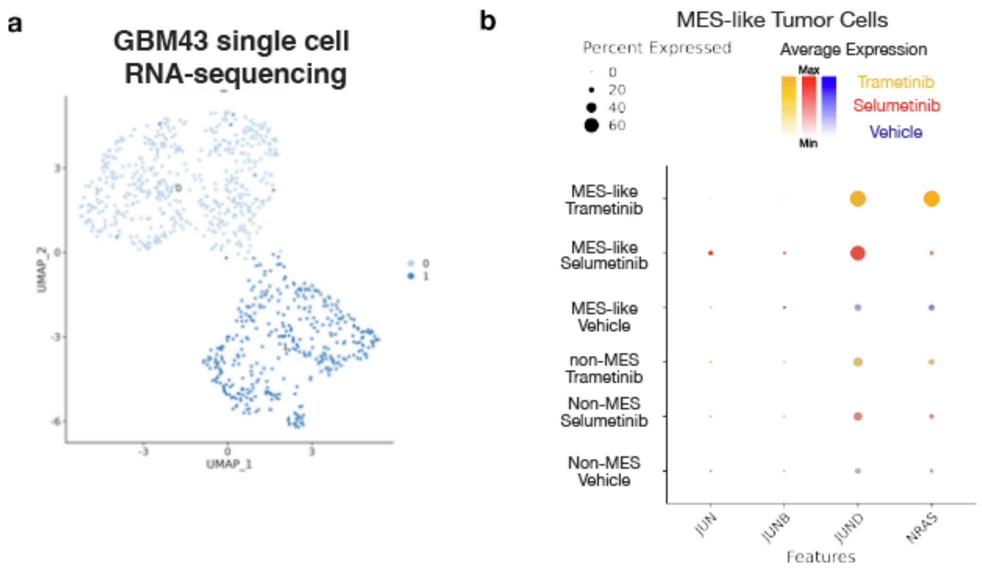

Supplementary Figure 13. scRNA-seq analysis of *NF1* mutant GBM43 neurospheres with MEK inhibition *in vitro*. a. scRNA-sequencing analysis of MEK inhibitor treated GBM43 neurospheres *in vitro* revealed two clusters. b. MEK inhibitor resistant MES-like cells exhibit Ras pathway activation with increased *JUN*D and *NRAS* expression.

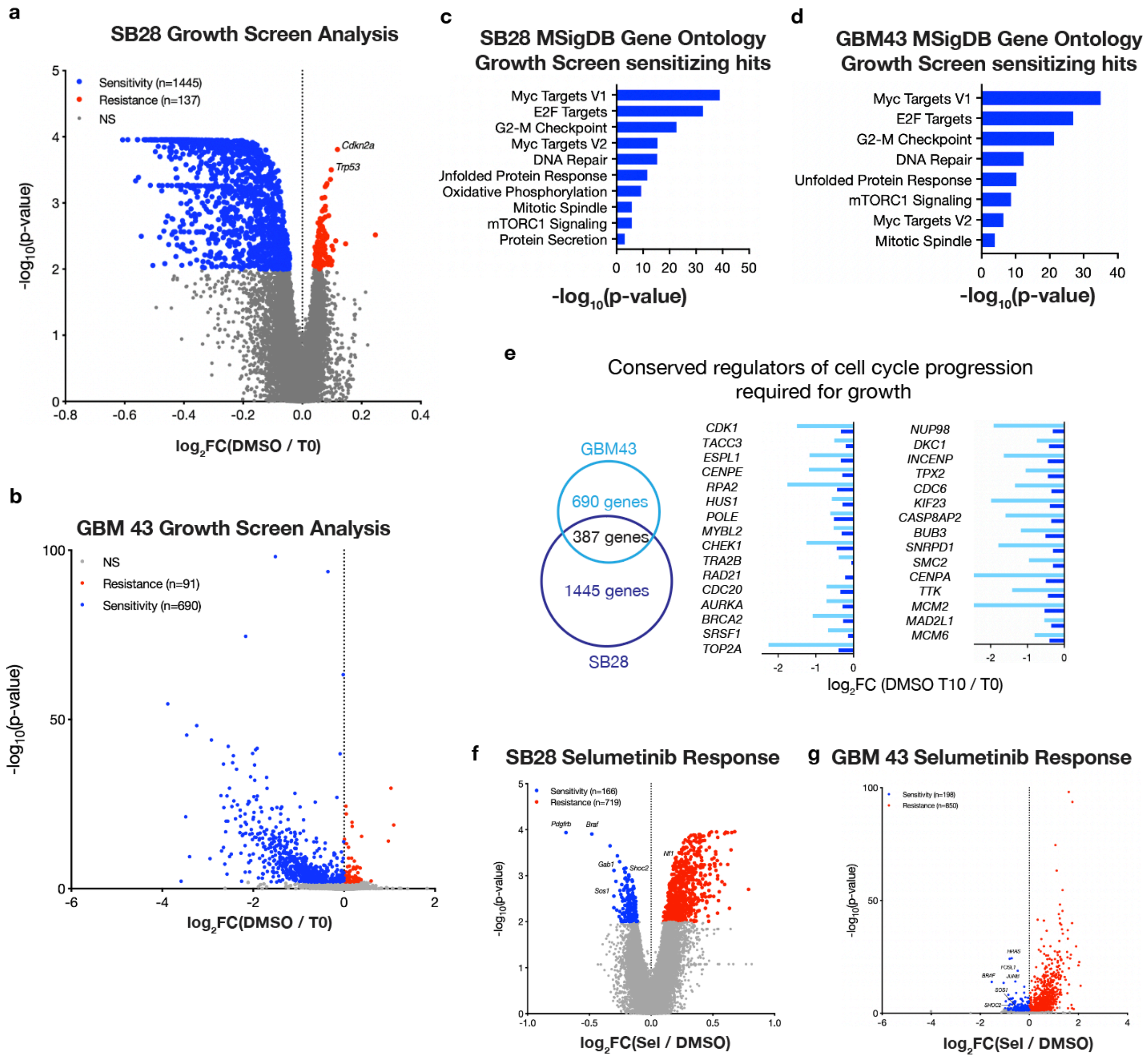

Supplementary Figure 14. Genome-wide CRISPRi screens for mediators of cell growth in SB28 or GBM43 glioblastoma cells reveals a conserved requirement for cell cycle genes.

a. Volcano plot of genes significantly mediating cell growth (Vehicle DMSO / T0) reveals 1445 genes underlying decreased growth and 137 genes underlying increased growth. Notably, *Cdkn2a* or *Tp53* repression are the top hits leading to increased cell growth. b. Volcano plot of genes significantly mediating cell growth (Vehicle DMSO / T0) reveals 690 genes underlying decreased growth and 91 genes underlying increased growth. c. Gene ontology analysis of genes leading to decreased glioblastoma cell growth when repressed reveals enrichment cell cycle gene sets (E2F Targets, G2-M Checkpoint, DNA Repair, Mitotic Spindle). d. Gene

ontology analysis of genes leading to decreased glioblastoma cell growth when repressed reveals enrichment cell cycle gene sets (E2F Targets, G2-M Checkpoint, DNA Repair, Mitotic Spindle) consistent with SB28 CRISPRi growth screen hits. e. Integrated analysis of genes significantly mediating cell growth (DMSO / T0) reveals a 31 gene conserved cell cycle network required for SB28 and GBM43 cell growth. f. Volcano plot of genes significantly mediating response to the MEK inhibitor selumetinib (Selumetinib / Vehicle DMSO control) reveals 166 genes mediating sensitivity and 719 genes mediating resistance in SB28 cells. g. Volcano plot of genes significantly mediating response to the MEK inhibitor selumetinib (Selumetinib / Vehicle DMSO control) reveals 198 genes mediating sensitivity and 850 genes mediating resistance in GBM43 cells.

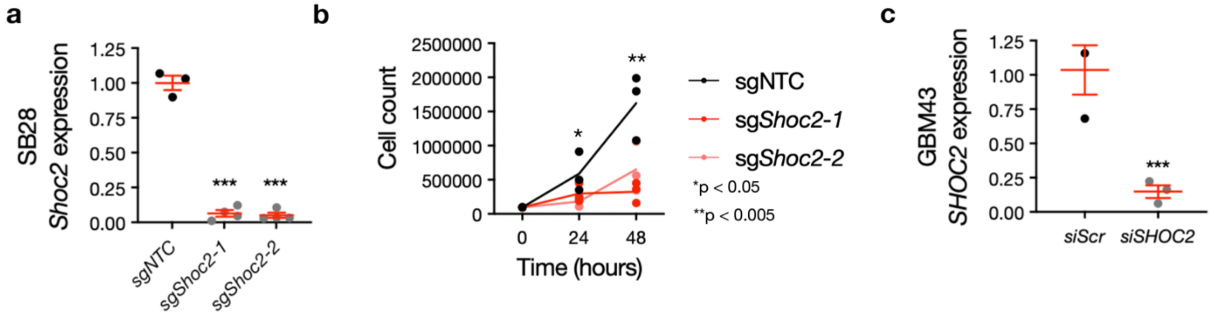

Supplementary Figure 15. Validation and analysis of *SHOC2* deficient glioblastoma cells.

a. qRT-PCR Validation of CRISPRi sg*Shoc2* SB28 cells. b. CRISPRi sg*Shoc2* SB28 cells show significantly decreased growth compared to sgNTC control SB28 cells. c. qRT-PCR validation of siRNA *siSHOC2* GBM43 cells.

a

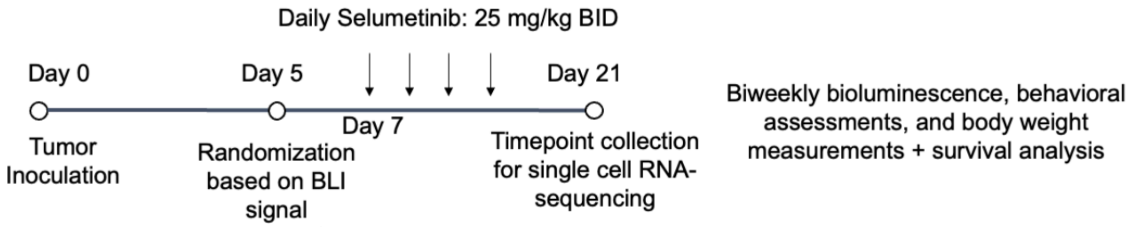

b

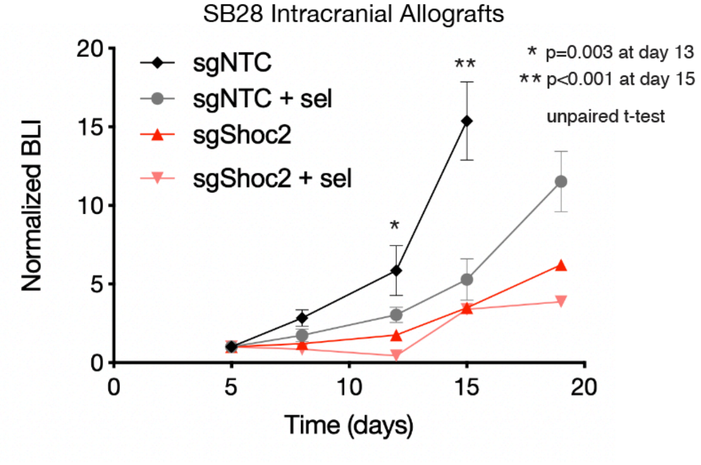

Supplementary Figure 16. Normalized bioluminescence (BLI) analysis following oral selumetinib treatment in CRISPRi control sgNTC or CRISPRi sg*Shoc2* SB28 allografts. a. Overview of mouse SB28 intracranial allograft experiment in immunocompetent host mice. b. BLI reveals *Shoc2* loss impedes tumor growth and is sufficient to further sensitize $NRAS^{G12V}$ mutant SB28 intracranial allografts to selumetinib (all statistical comparisons based on unpaired t-test).

Supplementary Tables.

Supplementary Table 1. Compiled variant list from the UCSF500 assay, a CLIA certified, capture based targeted DNA sequencing assay of 529 cancer-associated genes across *NF1* mutant, IDH-wildtype glioblastomas (n=186).

Supplementary Table 2. Propensity score matching to identify a *NF1* wildtype, IDH-wildtype glioblastoma cohort for baseline comparison.

Supplementary Table 3. Univariable Cox proportional hazards analysis using recurrently co-mutated genes across NF1 mutant and NF1 wildtype glioblastoma cohorts.

Supplementary Table 4. Univariable and multivariable Cox proportional hazards analysis using clinical factors and CDKN2A/B mutation across NF1 mutant glioblastomas.

Supplementary Table 5. Cluster marker gene list for snRNA-sequencing of human NF1 mutant glioblastomas (n=9).

Supplementary Table 6. Marker gene lists used to define glioblastoma non-tumor microenvironment populations.

Supplementary Table 7. Cluster marker gene list for snRNA-sequencing of Wang et al. human glioblastomas (n=53).

Supplementary Table 8. Cluster marker gene list for scRNA-sequencing of tumor cells from selumetinib (n=3) or vehicle treated (n=3) SB28 intracranial glioblastomas.

Supplementary Table 9. Differential gene expression analysis between selumetinib and vehicle treated tumor cells from SB28 intracranial glioblastomas.

Supplementary Table 10. Cluster marker gene list for scRNA-sequencing of GBM 43 cells treated with selumetinib, trametinib, or DMSO vehicle control.

Supplementary Table 11. Analysis of GBM43 CRISPRi screen comparing sgRNA enrichment between cells treated with selumetinib for 10 days compared to treated with vehicle for 10 days.

Supplementary Table 12. Analysis of SB28 CRISPRi screen comparing sgRNA enrichment between cells treated with selumetinib for 10 days compared to treated with vehicle for 10 days.
