# Supplementary material for: Tumor heterogeneity underlies clinical outcome and MEK inhibitor response in somatic NF1-mutant glioblastoma
Source: JCI Insight. 2025 Sep 23;10(18):e192658. doi: 10.1172/jci.insight.192658 (PMC12487855; doi:10.1172/jci.insight.192658)

Figure 4d, sgNTC conditions
In figure

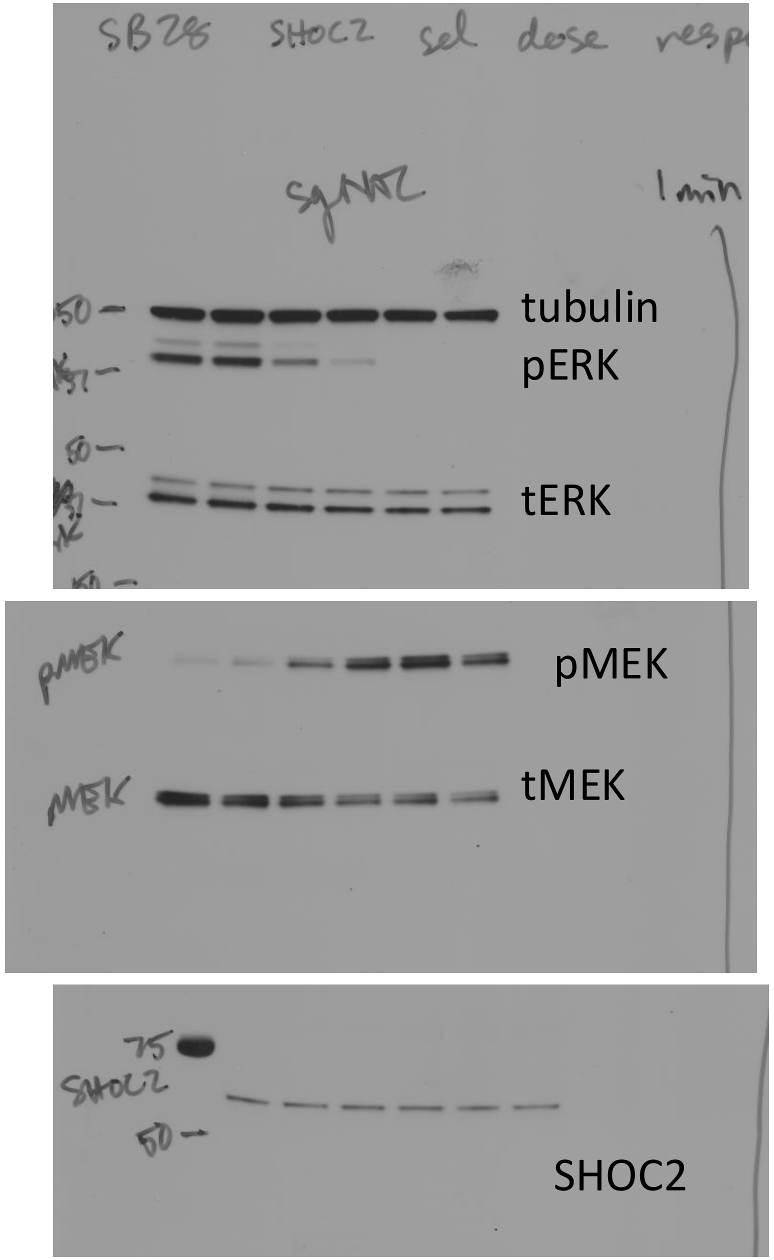

All exposures 30sec

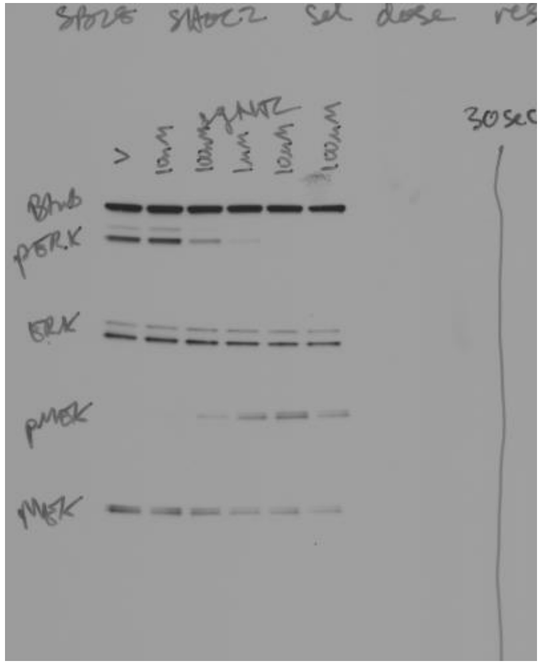

1 min

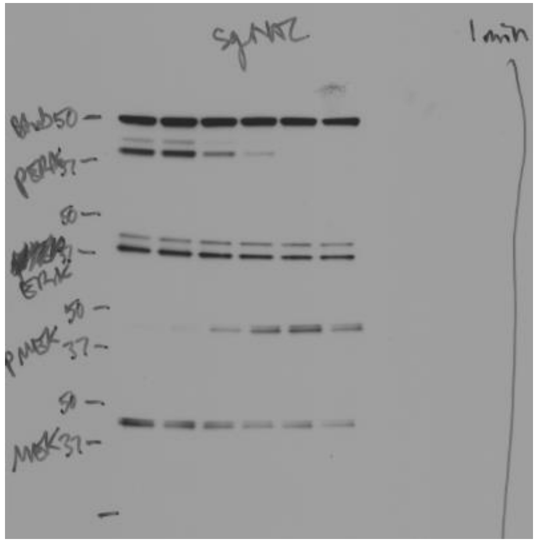

2 min

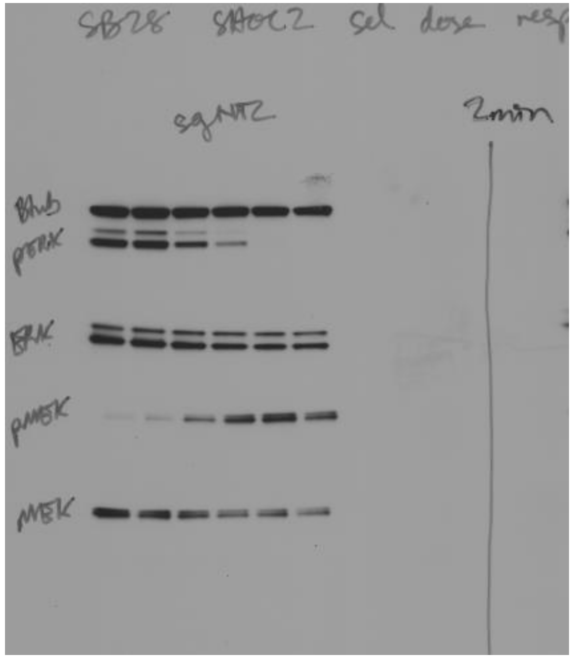

Figure 4d, sgSHOC2 conditions
In Figure

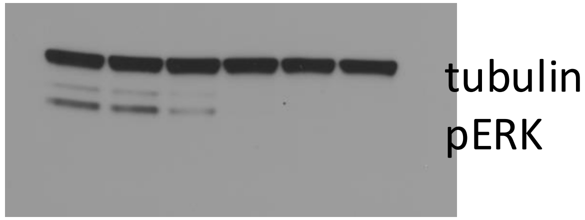

tubulin
pERK

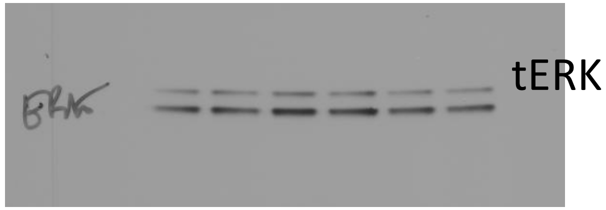

tERK

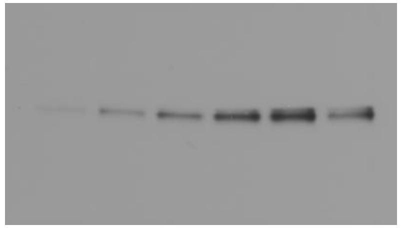

pMEK

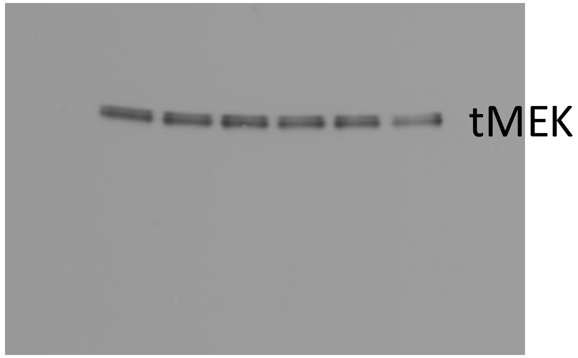

tMEK

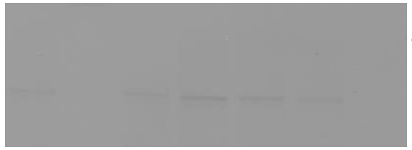

SHOC2

All exposures 30sec

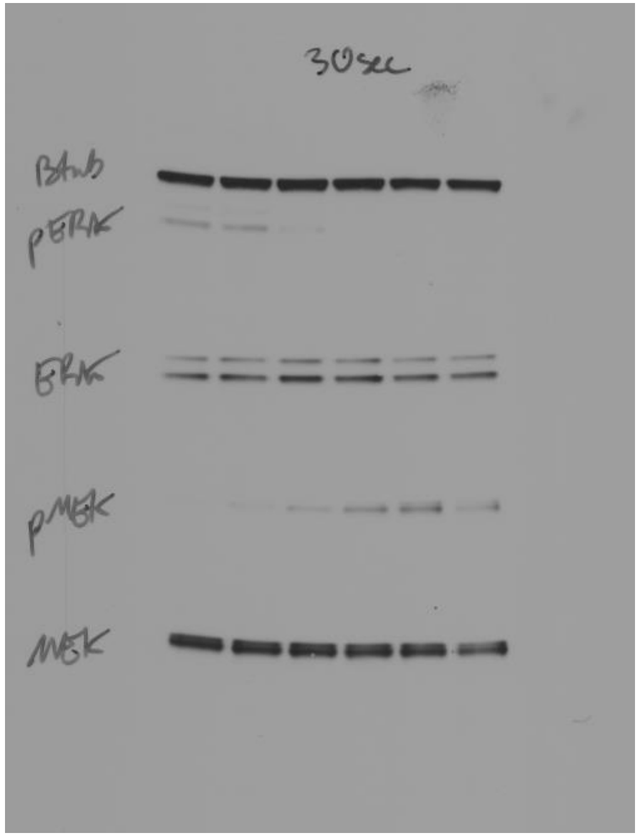

1 min

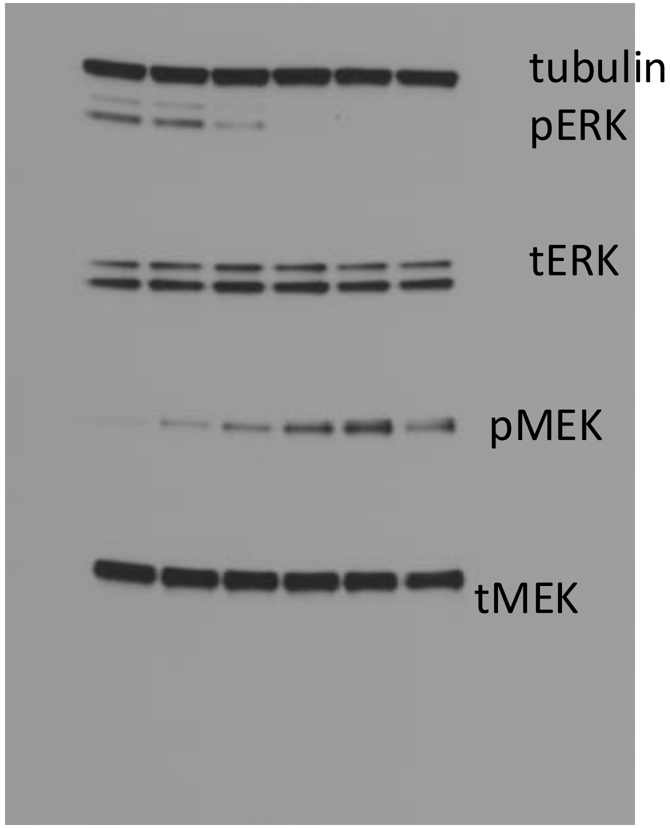

tubulin
pERK
tERK
pMEK
tMEK

Figure 4f, siSHOC2 in GBM43
In Figure

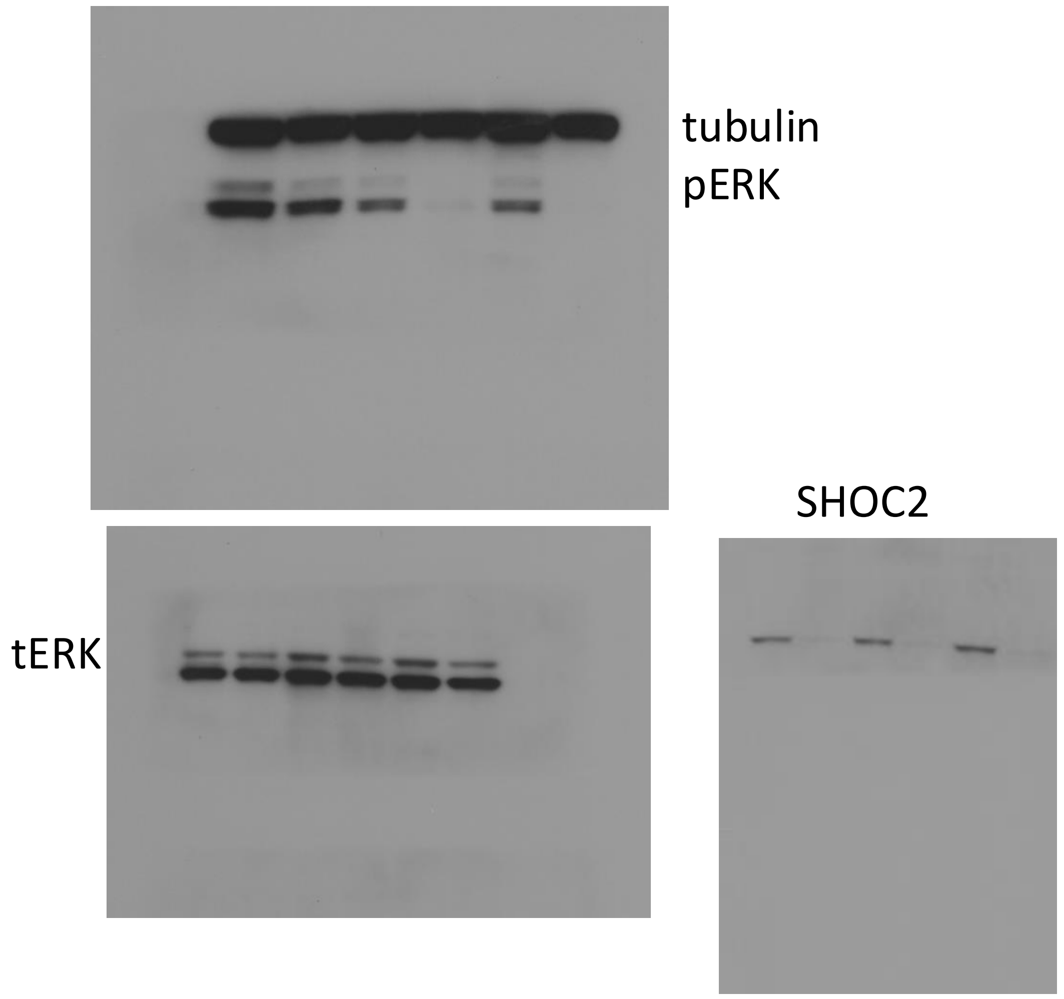

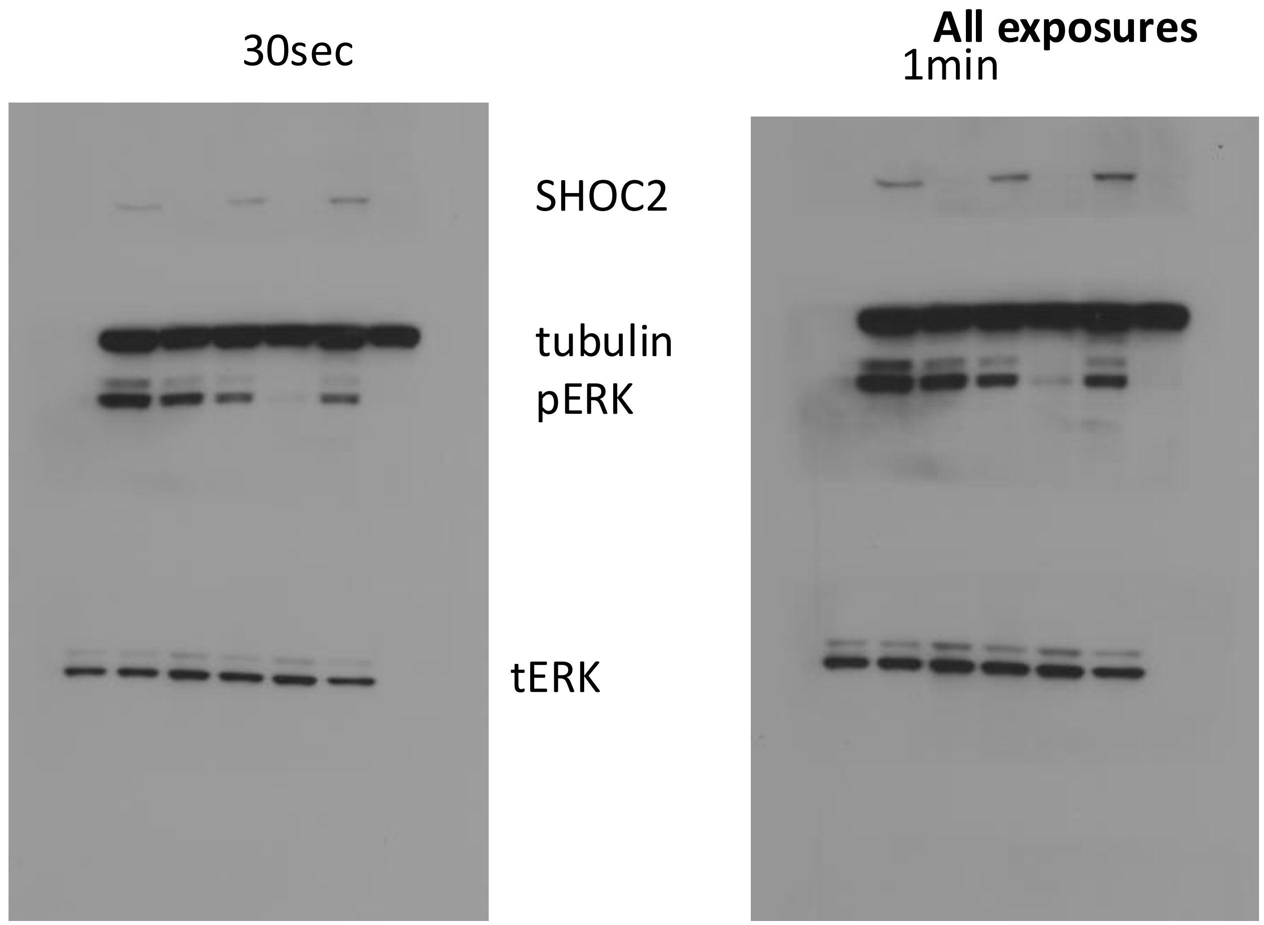

Supplement: Unedited blot and gel images [file jciinsight-10-192658-s010.pdf]
